# Associations between quality of life and duration and frequency of physical activity and sedentary behaviour: Baseline findings from the WALK 2.0 randomised controlled trial

**DOI:** 10.1371/journal.pone.0180072

**Published:** 2017-06-29

**Authors:** Gregory S. Kolt, Emma S. George, Amanda L. Rebar, Mitch J. Duncan, Corneel Vandelanotte, Cristina M. Caperchione, Anthony J. Maeder, Rhys Tague, Trevor N. Savage, Anetta Van Itallie, Nadeesha R. Mawella, Wei-Wen Hsu, W. Kerry Mummery, Richard R. Rosenkranz

**Affiliations:** 1 School of Science and Health, Western Sydney University, Sydney, NSW, Australia; 2 School of Human Health and Social Sciences, Central Queensland University, Rockhampton, QLD, Australia; 3 School of Medicine and Public Health, Priority Research Centre for Physical Activity and Nutrition, Faculty of Health and Medicine, University of Newcastle, Newcastle, NSW, Australia; 4 School of Health and Exercise Science, University of British Columbia, Kelowna, BC, Canada; 5 School of Health Science, Flinders University, Adelaide, SA, Australia; 6 School of Computing, Engineering and Mathematics, Western Sydney University, Sydney, NSW, Australia; 7 Department of Statistics, Kansas State University, Manhattan, KS, United States of America; 8 Faculty of Physical Education and Recreation, University of Alberta, Edmonton, AB, Canada; 9 Department of Food, Nutrition, Dietetics, & Health, Kansas State University, Manhattan, KS, United States of America; TNO, NETHERLANDS

## Abstract

While physical and mental health benefits of regular physical activity are well known, increasing evidence suggests that limiting sedentary behaviour is also important for health. Evidence shows associations of physical activity and sedentary behaviour with health-related quality of life (HRQoL), however, these findings are based predominantly on duration measures of physical activity and sedentary behaviour (e.g., minutes/week), with less attention on frequency measures (e.g., number of bouts). We examined the association of HRQoL with physical activity and sedentary behaviour, using both continuous duration (average daily minutes) and frequency (average daily bouts≥10 min) measures. Baseline data from the WALK 2.0 trial were analysed. WALK 2.0 is a randomised controlled trial investigating the effects of Web 2.0 applications on engagement, retention, and subsequent physical activity change. Daily physical activity and sedentary behaviour (duration = average minutes, frequency = average number of bouts ≥10 minutes) were measured (ActiGraph GT3X) across one week, and HRQoL was assessed with the ‘general health’ subscale of the RAND 36-Item Health Survey. Structural equation modelling was used to evaluate associations. Participants (N = 504) were 50.8±13.1 (mean±SD) years old with a BMI of 29.3±6.0. The 465 participants with valid accelerometer data engaged in an average of 24.0±18.3 minutes and 0.64±0.74 bouts of moderate-vigorous physical activity per day, 535.2±83.8 minutes and 17.0±3.4 bouts of sedentary behaviour per day, and reported moderate-high general HRQoL (64.5±20.0). After adjusting for covariates, the duration measures of physical activity (path correlation = 0.294, *p*<0.05) and sedentary behaviour were related to general HRQoL (path coefficient = -0.217, *p*<0.05). The frequency measure of physical activity was also significant (path coefficient = -0.226, *p*<0.05) but the frequency of sedentary behaviour was not significantly associated with general HRQoL. Higher duration levels of physical activity in fewer bouts, and lower duration of sedentary behaviour are associated with better general HRQoL. Further prospective studies are required to investigate these associations in different population groups over time.

## Introduction

Regular physical activity participation is associated with a range of positive health outcomes including improved mental health and a reduced risk of heart disease, type-2 diabetes, all-cause mortality, and some cancers [1,2]. In addition, physical activity participation has been shown to enhance health-related quality of life (HRQoL), a multidimensional measure of physical, functional, mental and social wellbeing [3]. In the general adult population, higher levels of physical activity have been associated with enhanced HRQoL outcomes [4–7]. In clinical populations, physical activity has also shown to be associated with HRQoL in survivors of colon cancer [8], adults with type-2 diabetes [9], and breast cancer survivors [10]. Despite this evidence, a substantial proportion of Australian adults fail to participate in levels of physical activity conducive to health benefits [11,12].

Time constraints are one of the most commonly reported barriers to being physically active [13–15]. Evidence suggests, however, that shorter bouts of physical activity (i.e., ≤10 minutes) accumulated across the day can provide similar health benefits to longer, sustained sessions of physical activity [16,17]. As such, the World Health Organization [1] encourages adults to accumulate aerobic activity in bouts of at least 10 minutes, and current Australian physical activity guidelines encourage accumulation of 150 to 300 minutes of moderate intensity or 75 to 150 minutes of vigorous intensity physical activity each week [18]. In many cases, these shorter bouts are likely to be more achievable for people with time constraints.

A growing body of evidence has also emerged suggesting an association between increasing sedentary behaviour and poor health outcomes, including increased risk of a range of chronic health conditions [19–22]. To reduce the deleterious effects of sedentary behaviour on health, research findings suggest that benefits can be gained from breaking up prolonged periods of sedentary time [21,23], and current Australian guidelines [18] suggest that adults minimise the amount of time they spend sitting, and break up long periods of sitting as often as possible.

Compared with physical activity, the association between sedentary behaviour and HRQoL is less established. Available evidence indicates that higher levels of self-reported leisure-time physical activity combined with lower levels of self-reported leisure-time sedentary behaviour [24] and self-reported sitting [25] are associated with better HRQoL (assessed using the Spanish version of the SF-36 [26] and a single item question, respectively). Similarly, when using self-reported screen time as an indicator of sedentary behaviour [27], adults reporting higher volumes of screen time paired with lower volumes of physical activity were more likely to report poorer self-reported HRQoL.

Most of the available evidence on the association between physical activity, sedentary behaviour, and HRQoL has been derived from studies using self-reported behavioural measures and have assessed physical activity and sedentary behaviour with duration measures (i.e., total time or energy expenditure). One particular study has examined the association between physical activity and HRQoL (measured by the EuroQol-5 Dimensions [28]) using both objective (ActiGraph accelerometers) and subjective (questionnaire) measures of physical activity [29]. Higher volumes of physical activity were associated with higher levels of HRQoL, however, the association between objectively measured physical activity and HRQoL was stronger than the association between subjectively measured physical activity and HRQoL [29]. Other studies have also demonstrated that higher levels of self-reported physical activity are associated with reduced coronary heart disease [30] and higher levels of objectively measured physical activity are associated with reduced cardiovascular disease risk [31], regardless of whether activity is accumulated in few long bouts, or a lot of short bouts.

Loprinzi and Davis [32] examined the association between objectively measured bout (≥10 minutes in duration) and non-bout (<10 minutes in duration) moderate-vigorous physical activity (MVPA) and HRQoL (measured using the CDC HRQoL [33]) in 5,530 American adults aged ≥20 years. Higher levels of participation in both bout and non-bout physical activity was associated with higher HRQoL, suggesting that MVPA, regardless of how it was accrued can enhance HRQoL. Differences between bout and non-bout MVPA have also been examined in relation to other health outcomes such as risk of all-cause mortality [34] and cardiovascular disease biomarkers [35]. Loprinzi [34] found that engaging in more frequent bouts of at least 30 minutes of MVPA each day, in comparison to engaging in longer, less frequent bouts across the week, was a stronger predictor of C-reactive protein, an independent predictor of chronic disease risk. In another study, Loprinzi [36] examined the joint associations between objectively-measured physical activity and sedentary behaviour with HRQoL, as measured by the CDC HRQoL-4 [37], and found that the duration measure of total daily sedentary behaviour was not independently associated with HRQoL.

As evidence continues to emerge on the benefits of shorter bouts of physical activity [31,38], and the deleterious effect of prolonged bouts of sedentary time [19,39,40], it is important to consider whether the duration and frequency of these behaviours contribute differently to health outcomes such as HRQoL. Existing evidence suggests that both duration and frequency measures of objectively measured MVPA are associated with HRQoL [34]; and that duration measures of sedentary behaviour are not independently associated with HRQoL [36]. There are no studies to our knowledge, however, that have compared duration and frequency measures of objectively measured sedentary behaviour with HRQoL.

Using baseline data from the WALK 2.0 trial [41], the purpose of this study was to examine the association of HRQoL with physical activity and sedentary behaviour, using both continuous duration (average daily minutes) and frequency measures (average daily number of bouts ≥10 min).

## Materials and methods

This study utilised the baseline data from WALK 2.0, a trial investigating the effects of Web 2.0 applications on engagement, retention, and subsequent physical activity behaviour change in a web-based physical activity intervention [41].

### The WALK 2.0 trial

The WALK 2.0 trial has been described in detail elsewhere [41,42]. Briefly, WALK 2.0 is a three-arm randomised controlled trial (RCT) comparing the effectiveness of two web-based interventions with a paper-based logbook physical activity intervention on a range of outcomes including: physical activity; HRQoL; anthropometric measures; website usage, engagement, and retention; internet self-efficacy, psychosocial variables, and system usability. Outcomes are assessed at baseline, and at 3, 12, and 18 months. The WALK 2.0 trial was registered prospectively with the Australian New Zealand Clinical Trials Registry (ACTRN12611000157976)–see https://www.anzctr.org.au/Trial/Registration/TrialReview.aspx?id=336443&isReview=true.

Participants were recruited for WALK 2.0 across 2 sites in Australia (South Western Sydney and Central Queensland). Recruitment was primarily through personalised invitation letters to an extract of individuals selected randomly from the Australian Electoral Commission electoral roll. Other forms of recruitment were through advertisement in local print media, email messages to university email lists, and through those who had registered with one of the partner universities as being interested in research participation. Participants were deemed eligible for the trial if they were over 18 years of age, had access to the Internet, reported doing less than 30 minutes of MVPA on 5 or more days of the week [43], did not have an existing medical condition that would contraindicate physical activity (as assessed by the Physical Activity Readiness Questionnaire, PAR- Q [44]), and had not ever been a member of the existing 10,000 Steps program [45].

**Fig 1:**
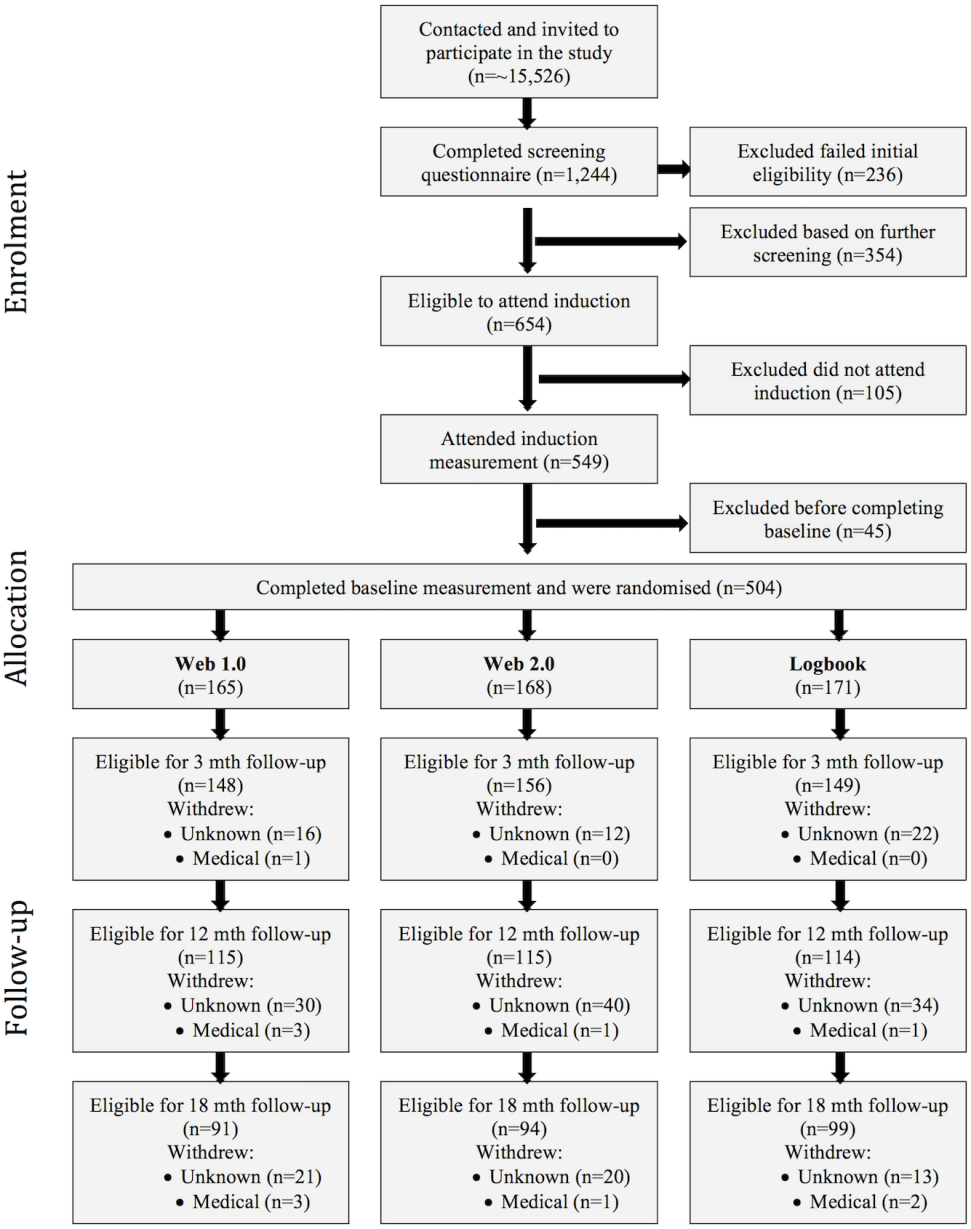
Flow of trial protocol.

Participants attended an induction session where they were fitted with an ActiGraph GT3X activity monitor (ActiGraph, Pensacola USA) to measure physical activity over 7 days. They then attended a baseline measurement session where all remaining outcome measures were administered. Following completion of baseline measures, participants were then randomly assigned to one of 3 trial arms: Web 1.0, Web 2.0, or logbook (Fig 1). Those in the Web 1.0 group participated in the existing 10,000 Steps program [45] designed to increase physical activity through the use of a pedometer, an online step log, individual self-monitoring features and electronic educational materials. Those in the Web 2.0 group had access to a newly developed website (WALK 2.0) that maintained core 10,000 Steps website data management functionality, and also provided Web 2.0 patterns of interaction to establish user-to-user engagement through social networking capabilities of status posting, activity streams, virtual walking groups, personal blogs. Participants in the logbook group were provided with a paper-based logbook and key written messages available through the other two intervention arms (e.g., increasing opportunities for physical activity, instruction on goal setting).

Ethical approval for the WALK 2.0 trial was granted by the Human Research Ethics Committees of Western Sydney University (Reference Number H8767) and Central Queensland University (H11/01-005). All participants gave consent to participate.

### Participants

Participants for this study were all those enrolled in the WALK 2.0 trial at baseline (N = 504). Participants were 50.8 ± 13.1 (mean ± SD) years old, had a BMI of 29.3 ± 6.0, were mostly female (*n* = 328, 65.1%), and had completed a certificate, diploma, or university degree (*n* = 364, 72.2%). Participants were recruited between March 2012 and July 2013, and 18-month follow-up took place between September 2013 and January 2015.

### Measures

The duration and frequency measures of physical activity and sedentary behaviour were assessed objectively using the ActiGraph GT3X activity monitor. Participants were asked to wear the monitors for 7 consecutive days during waking hours, except for water-based activities or contact sports. The ActiGraph was affixed to an elastic belt to be worn on the wait with the ActiGraph positioned over the right hip. Units were initialised to collect triaxial acceleration and step count data using 1 second epochs. Prior to analyses the data were aggregated to 60 second epochs using Actilife software 6.6.3. Based on activity counts per minute, a customised Microsoft Excel macro, written in visual basic, was used to provide daily measures of MVPA (more than 1951 counts/min) or sedentary (less than 100 counts/min) behaviour [46,47], bouts of MVPA, bouts of sedentary time and wear time. Non-wear time was defined as 60 minutes of consecutive zero counts and included a 2 minute spike tolerance of 50 counts per minute of movement. Valid wear time was defined as a minimum of 10 hours of wear time on at least 5 days in the 7 day period, and was required to be included in the analysis. The average daily time spent in each of these activities was used as the duration measures of MVPA and sedentary behaviour. A bout was classified as any consecutive 10-minute period of MVPA or sedentary behaviour, and the average number of daily bouts was used as the frequency measures of MVPA and sedentary behaviour.

HRQoL was assessed using the 5-item ‘general health’ subscale of the RAND 36-Item Health Survey (RAND-36), a license-free instrument developed from the original SF-36 Medical Outcomes Study survey [48]. Although both instruments contain the same survey items, the scoring algorithms for the body pain and general health subscales are slightly different in the RAND 36 [48]. This has been discussed in detail elsewhere [42], but briefly, all items are scored on a scale of 0 to 100, with a higher score indicating a more favourable health state. Missing data are excluded, and the scores are averaged for each construct to generate 8 separate scores. The RAND 36 has been validated for measuring HRQoL in Australian populations [49] and has been shown to be suitable for use in general populations [50, 51].

### Data analyses

Descriptive analyses were completed and presented as means and standard deviations (SD) for continuous variables and as frequencies and proportions for categorical data. The duration and frequency measures of sedentary behaviour were highly correlated, as were the duration and frequency measures of physical activity, so regression modelling was not appropriate for this analysis. To account for multicollinearity, path analysis, a special case of structural equation modelling [52, 53] was used to model the complex associations between duration and frequency measures of sedentary behaviour and physical activity and HRQoL. Due to positive skewness, log transformations of physical activity minutes and bouts were performed, and the log-transformed variables were used in the path analysis to evaluate whether the duration and frequency measures of physical activity and sedentary behaviour were associated with general HRQoL. This analysis also included covariates of age, gender, BMI, level of education, and activity monitor wear time. To evaluate the overall performance of this analysis, Chi square test statistic, Root Mean Square Error of Approximation (RMSEA) index, Goodness of Fit Index (GFI), and the Bentler Comparative Fit Index (CFI) [54] were adopted as model fit indices. Typically, a Chi square test with a *p*-value of >0.05, RMSEA with a value close to zero, and CFI (or GFI) with a value close to one indicate a good model fit [55, 56]. In addition to several overall model fit indices, we also examined the variance-covariance matrix to evaluate the model fit. Observation of the variance-covariance matrix of the path analysis facilitates further understanding of the relationships among the exogenous variables and provides additional information about the data. Path analysis was performed using Statistical Analysis Software (SAS) 9.4 with the procedure CALIS, and path coefficients were evaluated for statistical significance with a *p*-value of <0.05. We performed *post hoc* power analysis based on root-mean-square error (RSME), an index to evaluate the overall model fit [57]. The posterior power was 0.942 with our data, suggesting that the study was adequately powered to detect a poor model fit (i.e., RMSE>0.05).

### Results

Descriptive statistics and correlations of general HRQoL and the duration and frequency measures of physical activity and sedentary behaviour are shown in Table 1. Valid activity monitor data were available for 465 participants, who engaged in an average of 24.0 ± 18.3 minutes and 0.64 ± 0.74 bouts of MVPA per day, an average of 535.2 ± 83.8 minutes and 17.0 ± 3.4 bouts of sedentary behaviour per day, mean wear time was 867.2 ± 73.7 minutes per day, and reported moderate-high levels of general HRQoL (64.5 ± 20.0) on a 0–100 scale.

**Table 1:** Descriptive statistics and correlations of general health-related quality of life, and duration and frequency measures of physical activity and sedentary behaviour.

Variable	M	SD	2.	3.	4.	5.
1. General Health	64.53	19.99	.05	.01	-.09	-.06
2. Physical Activity (min/day)	23.97	18.26	—	.86*	-.11*	-.07
3. Physical Activity (bouts/day)	0.64	0.74		—	-.06	-.02
4. Sedentary Behaviour (min/day)	535.20	83.82			—	.88*
5. Sedentary Behaviour (bouts/day)	16.97	3.43				—

*N* = 465

*p < .05

Spearman correlation coefficients.

**Fig 2:**
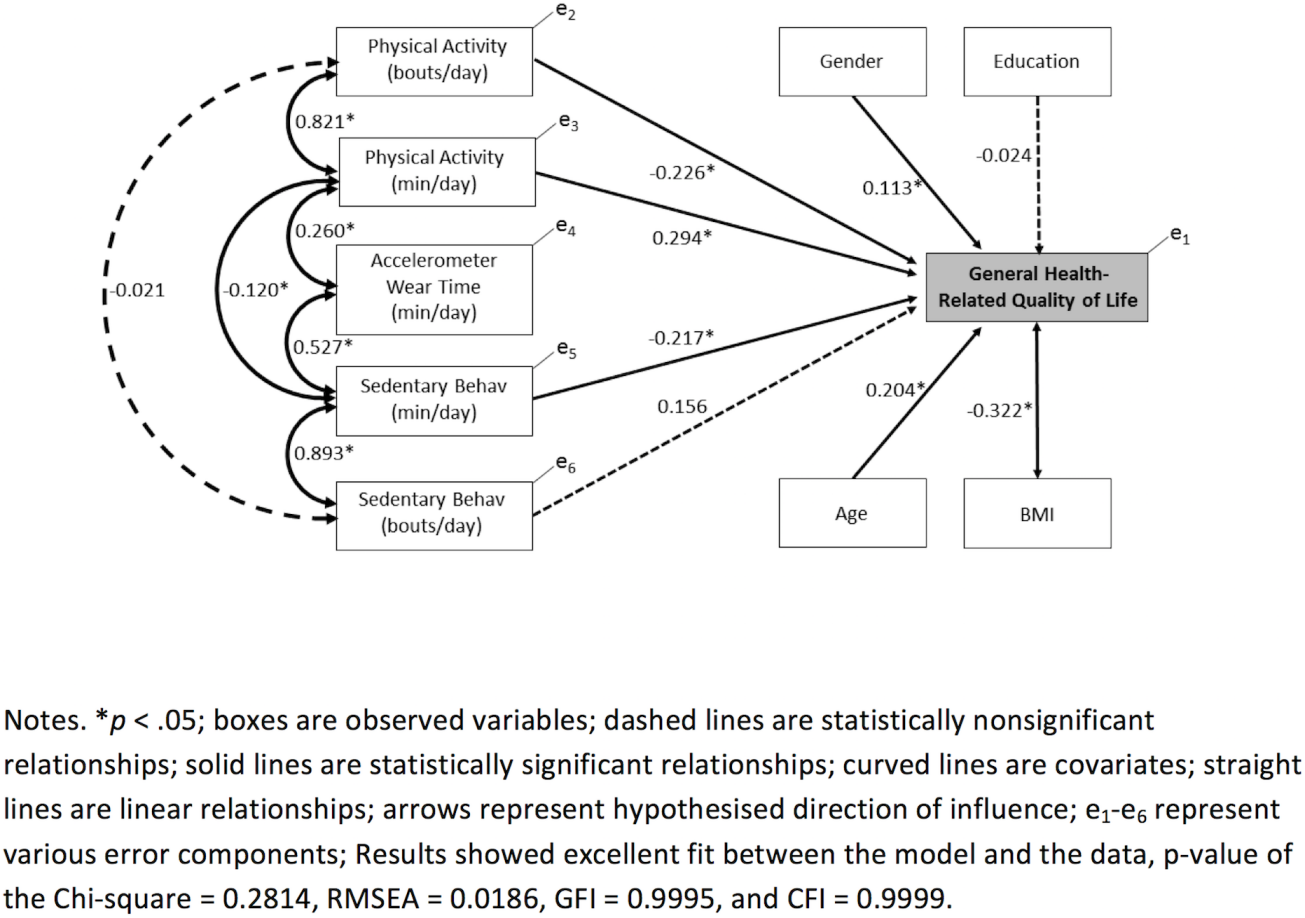
Path analysis model illustrating the observed relationships between physical activity, sedentary behaviour, and general health-related quality of life.

Results of the path analysis are provided in Fig 2. The association between the duration (average daily minutes) and the frequency (average daily number of bouts) measures of physical activity was significant, with an estimated path coefficient 0.821 (*p*<0.05). Likewise, the duration and frequency measures of sedentary behaviour were positively correlated with an estimated path coefficient 0.893 (*p*<0.05).

The duration measure (average daily minutes) of physical activity was positively related to general HRQoL (path coefficient = 0.294, *p*<0.05) after adjusting for covariates of age, gender, BMI, level of education, and activity monitor wear time, suggesting people with more average daily physical activity (min/day) had a higher general HRQoL score. In contrast, the physical activity bouts measure was negatively related to general HRQoL (path coefficient = -0.226, *p*<0.05) after adjusting for covariates.

The duration measure (average daily minutes) of sedentary behaviour was negatively related to general HRQoL (path coefficient = -0.217, *p*<0.05) after adjusting for covariates of age, gender, BMI, level of education, and activity monitor wear time, suggesting people with more average daily sedentary behaviour (min/day) had a lower general HRQoL score. After adjusting for covariates, the frequency measure of sedentary behaviour (path coefficient = 0.156, *p*<0.05) was not significantly associated with general HRQoL (Fig 2).

Additionally, the duration measures of physical activity and sedentary behaviour were negatively correlated with estimated path coefficient of -0.120 (*p*<0.05), indicating that a longer duration of physical activity was coupled with a shorter duration of sedentary behaviour, and that a shorter duration of physical activity was coupled with a longer duration of sedentary behaviour. The duration measures of physical activity and sedentary behaviour were also positively associated with activity monitor wear time (min/day) with a path coefficient of 0.260 (*p*<0.05) and 0.527 (*p*<0.05), respectively.

Overall, general HRQoL was associated with other important covariates. A positive association with age (path coefficient = 0.204, *p*<0.05) indicated older people reported higher general HRQoL scores. Females had higher general HRQoL scores compared to males, as gender (path coefficient = 0.113, *p*<0.05) was positively correlated with general HRQoL. BMI was negatively associated with general HRQoL (path coefficient = -0.322, *p*<0.05), indicating that people with a higher BMI reported lower general HRQoL scores. For the model fit indices, the *p*-value of the Chi-square test statistic was 0.2814, the RMSEA was 0.0186 with 90% confidence interval (0, 0.1263), GFI was 0.9995, and the CFI was 0.9999, which collectively suggests excellent fit between the model and the data.

## Discussion

The aim of this study was to examine the association of HRQoL with physical activity and sedentary behaviour, and to explore this association using both continuous duration (i.e., average daily minutes) and frequency (i.e., number of bouts ≥10 min) measures of the behaviours.

After adjusting for a range of covariates, there was a significant positive association between physical activity duration and general HRQoL, and also an inverse association between the duration measure of sedentary behaviour and general HRQoL, as those with more average physical activity and less daily sedentary behaviour had higher HRQoL scores. Additionally, the physical activity bouts variable was significantly inversely related to HRQoL, suggesting that for a given level of physical activity duration, being active in fewer bouts was associated with better health. This result reinforces current physical activity guidelines [1, 18] that are based on the findings of previous studies that have examined the association between physical activity and HRQoL [24,25,29,58]. For example, higher levels of participation in objectively measured bout and non-bout physical activity was shown to be associated with higher HRQoL [32] and both objective and subjective duration measures of physical activity were associated with better HRQoL [29] in adults aged 40–60 years. Like our study, physical activity was objectively measured using validated activity monitors in those previous studies. In the study by Anokye and colleagues [29], however, participants were categorised as ‘physically active’ (i.e., achieving a minimum 90 minutes of moderate physical activity per week) or ‘not physically active’ across a range of physical activity categories. By dichotomising the physical activity data, the extent of the variance in physical activity may be underestimated [59] and it can be difficult to compare these findings to those in the current study. Despite some heterogeneity of measurement tools used to assess HRQoL, our results show concordance with previous literature on the independent contribution of both duration and bouts of physical activity. In the current study, the general health subscale of the RAND 36 was used, while other studies have used instruments including the CDC HRQoL-4 [58], and the EuroQol-5 Dimensions [29] to measure HRQoL.

The small positive association between the bouts measure of sedentary behaviour and general HRQoL, however, was not significant, suggesting that the duration measure of sedentary behaviour may be more influential on general HRQoL. This finding is consistent with other studies that have examined the association between self-reported duration of sedentary behaviour and HRQoL [24,25,27], but diverges from recent literature that emphasises the importance of breaking up sedentary time for decreasing health risks [60]. It could be that self-report measures are less accurate in assessing sedentary behaviour, and as such, influence findings. Interestingly, the current finding differed from the results of an earlier study that examined the joint associations of objectively-measured physical activity and sedentary behaviour with HRQoL [36]. After adjusting for covariates, Loprinzi found that continuous sedentary behaviour was not independently associated with HRQoL [36].

### Implications for practice

These findings indicate that both higher physical activity and lower duration of sedentary behaviour are associated with improved HRQoL. Both duration and frequency measure of MVPA are associated with HRQoL. Combined with other evidence on the impact of sedentary behaviour on health outcomes, particularly for those not engaging in MVPA, reducing sedentary behaviour may be an important intervention target. However given the limitations of this research, further studies are needed to replicate these observations and identify potential mechanisms.

### Implications for research

The use of path analysis allows researchers to test complex models over time, and allows for comparison with similarly complex models. The use of path analysis is a novel approach and should be used for future research in this area. Although our hypothesised model showed excellent fit to the data in this sample, such a model should be tested in longitudinal studies and other populations. Further research should consider both duration and frequency measures of sedentary behaviour and physical activity.

### Strengths and limitations

Many of the studies that have examined the association between physical activity, sedentary time and HRQoL have focussed on older adults [4,24], or adults with chronic conditions [8–10]. In a systematic review of the literature, Bize et al. [4] recognised that older adults and individuals with chronic disease often present with particular challenges and needs related to their HRQoL profile, and identified a need for further evidence regarding the benefits of physical activity on HRQoL in healthy populations. This study builds upon the earlier findings of Loprinzi [58] in examining key differences between duration and frequency measures of physical activity and HRQoL, and adds to the limited body of evidence examining the association between sedentary behaviour and HRQoL.

The results of the current study should be interpreted in light of the potential limitations. The eligibility criteria used to assess participants’ eligibility to enrol in the study purposefully excluded those who were engaging in more than 30 minutes of physical activity on five or more days of the week [61,62]. This may have restricted the true physical activity variability that exists within a general population, and may have influenced the findings of the study. The cross-sectional nature of the analysis only provides evidence of associations between physical activity, sedentary behaviour, and HRQoL, and cannot determine causal relationships. Although this study is cross-sectional, unlike many of the studies included in an earlier systematic review of the literature [4], this study used objectively measured data on physical activity and sedentary behaviour. This is an important strength of the current study, as objective measures of physical activity and sedentary behaviour are less prone to measurement error and recall bias, and are often more representative of an individual’s true physical activity and sedentary behaviour. One potential limitation of the use of objectively measured data is that we could not delineate domain-specific physical activity and sedentary behaviour, and as such, the amount of time spent in specific domains of physical activity and sitting time (e.g., leisure, occupational, transportation, socialising) could not be established. A final potential limitation relates to the lack of consensus on what the minimum duration should be to be considered a “bout” of physical activity or sedentary behaviour. Whilst in this study we used 10 minutes, further research is still needed to establish whether this length of bout is the most appropriate for both physical activity and sedentary behaviour. It could be that examining pattern distribution of bouts of physical activity and sedentary behaviour provides a more robust measure by which to relate to health outcomes [63].

The use of path analysis (a special case of structural equation modelling or SEM) is a further strength of this study, as this particular modelling procedure addresses issues of multicollinearity, and allows for identification of direct and indirect effects. SEM can be used to evaluate the underlying association structure of variables, especially for complex models [52, 53]. This approach often requires a large sample size for a precise model estimation, because a regular SEM usually includes latent variables in the model, generating more parameters [57]. With the sample size of this study, it could have been unsatisfactory to use a regular latent structure SEM that involved more parameters. In this study, we adopted path analysis, which did not require latent variables in the model. Thus, the path analysis was well suited to address our research questions with available data, and to provide more reliable statistical inferences.

## Conclusions

Previous evidence suggests that duration and frequency measures should be considered as different components of behaviour, rather than as corresponding or equivalent measures of the same behaviour [64]. This study is one of the first to examine the association of HRQoL with objectively-measured physical activity and sedentary behaviour, using both continuous duration (total minutes) and frequency (number of bouts ≥10 min) measures of these behaviours. There was a significant association between both the frequency of physical activity, the duration measures of physical activity and sedentary behaviour and the outcome of HRQoL. There was no association between the frequency measure of sedentary behaviour and HRQoL. There is a need for further prospective research using objective duration and frequency measures of physical activity and sedentary behaviour to examine these relationships in larger cohorts over time.

## Supporting information

S1 File Consort checklist. (DOC)

S2 File Trial protocol–Ethics committee approved. (PDF)
