# Supplementary material for: Associations between quality of life and duration and frequency of physical activity and sedentary behaviour: Baseline findings from the WALK 2.0 randomised controlled trial
Source: PLoS One. 2017 Jun 29;12(6):e0180072. doi: 10.1371/journal.pone.0180072 (PMC5491114; doi:10.1371/journal.pone.0180072)
Supplement: S2 File — (PDF) [file pone.0180072.s002.pdf]

National Ethics Application Form

Version 2008 - V2.0

Proposal title: **WALK 2.0: Investigating the internal and external validity of Web 2.0 applications in promoting physical activity**

For submission to:

Central Queensland University's Human Research Ethics Committee (EC00158)

University of Western Sydney Human Research Ethics Committee (EC00314)

Name: **Prof Gregory Kolt**

Address: **University of Western Sydney**
Locked Bag 1797
Penrith South DC NSW 1797
Australia

Contact: (Bus) **+61(2) 4620 3747**
(AH) **-**
(Mob) **-**
(Fax) **+61(2) 46203710**

Proposal status: **Complete**

Proposal description:

More than half of the Australian population do not meet the levels of physical activity recommended to achieve health benefits. The Internet has become an increasingly important communication tool and Internet based physical activity interventions which include innovative technology have the potential to reach large groups of individuals and contribute to the promotion of physical activity and behaviour change. Each year there is a proliferation of health-related and health-promotion websites, yet virtually no work has been done to study the utility and effectiveness of these population-targeted websites. This study will develop a website featuring Web 2.0 applications and evaluate the efficacy and utility of the website to promote physical activity in comparison to a conventional (Web 1.0) physical activity promotion website and a control group. The study utilises two methods to achieve the overall aim of the project - a randomised control trial (RCT) and a ‘real world' ecological trial (ET). The RCT will investigate the efficacy, adherence and behaviour change in a rigorous, controlled setting with high internal validity. The ET takes advantage of the unique ‘real world' sample available to the research team through the existing 10,000 Steps website to evaluate engagement by users from the broader community. We expect that the current proposal will provide an increased understanding of the benefits of newer web-based technologies and applications in engaging and retaining participants on web-based physical activity promotion sites. This work has the potential to improve health outcomes from increased engagement/retention in physical activity.

Previously submitted to:

Administrative Section

1. TITLE AND SUMMARY OF PROJECT

1.1. Title

1.1.1 What is the formal title of this research proposal?

WALK 2.0: Investigating the internal and external validity of Web 2.0 applications in promoting physical activity

1.1.2 What is the short title / acronym of this research proposal (if applicable)?

Walk 2.0

1.2. Description of the project in plain language

1.2.1 Give a concise and simple description (not more than 400 words), in plain language, of the aims of this project, the proposal research design and the methods to be used to achieve those aims.

More than half of the Australian population do not meet the levels of physical activity recommended to achieve health benefits. The Internet has become an increasingly important communication tool and Internet based physical activity interventions which include innovative technology have the potential to reach large groups of individuals and contribute to the promotion of physical activity and behaviour change. Each year there is a proliferation of health-related and health-promotion websites, yet virtually no work has been done to study the utility and effectiveness of these population-targeted websites. This study will develop a website featuring Web 2.0 applications and evaluate the efficacy and utility of the website to promote physical activity in comparison to a conventional (Web 1.0) physical activity promotion website and a control group. The study utilises two methods to achieve the overall aim of the project - a randomised control trial (RCT) and a 'real world' ecological trial (ET). The RCT will investigate the efficacy, adherence and behaviour change in a rigorous, controlled setting with high internal validity. The ET takes advantage of the unique 'real world' sample available to the research team through the existing 10,000 Steps website to evaluate engagement by users from the broader community. We expect that the current proposal will provide an increased understanding of the benefits of newer web-based technologies and applications in engaging and retaining participants on web-based physical activity promotion sites. This work has the potential to improve health outcomes from increased engagement/retention in physical activity.

2. RESEARCHERS / INVESTIGATORS

2.2. Principal researcher(s) / investigator(s)

2.2.0 How many principal researchers / investigators are there? 7

2.2.1. Principal researcher / investigator 1

2.2.1. Name and contact details

Name: Prof Gregory Kolt

Address: School of Biomedical and Health Sciences
University of Western Sydney
Locked Bag 1797
Penrith South DC NSW 1797

Organisation: University of Western Sydney

Area: School of Biomedical and Health Sciences

Position: Head of School

Contact: (Bus) +61 (0)2 4620 3590 (AH) -
(Mob) - (Fax) +61 (0)2 4620 3710

Email: g.kolt@uws.edu.au

2.2.2... Summary of qualifications and relevant expertise NS 4.8.7 NS 4.8.15
PhD qualified. Many years of experience in physical activity and health promotion research.

2.2.2... Please declare any general competing interests
Nil.

2.2.2... Name the site(s) for which this principal researcher / investigator is responsible.
Greater Western Sydney and Central Queensland

2.2.3 Describe the role of the principal researcher / investigator in this project.
Oversee the project scientific direction and delivery and work with the research team to determine the nature of the data collected.

2.2.4 Is the principal researcher / investigator a student? No

2.2.1. Principal researcher / investigator 2

2.2.1. Name and contact details

Name: Prof Anthony Maeder

Address: School of Computing and Mathematics
University of Western Sydney
Locked Bag 1797
Penrith South DC NSW 1797

Organisation: University of Western Sydney

Area: School of Computing and Mathematics

Position: Professor of Health Informatics

Contact: (Bus) +61(0)2 46203462 (AH) -
(Mob) - (Fax) +61 (0)2 4620 3075

Email: a.maeder@uws.edu.au

2.2.2... Summary of qualifications and relevant expertise NS 4.8.7 NS 4.8.15
PhD qualified. Strong research and applied background in health informatics.

2.2.2... Please declare any general competing interests
Nil.

2.2.2... Name the site(s) for which this principal researcher / investigator is responsible.
Greater Western Sydney and Central Queensland

2.2.3 Describe the role of the principal researcher / investigator in this project.
Work with the research team on scientific direction and development of the the web-based applications.

2.2.4 Is the principal researcher / investigator a student? No

2.2.1. Principal researcher / investigator 3

2.2.1. Name and contact details

Name:	Dr Corneel Vandelanotte
Address:	Institute for Health and Social Science Research<br>CQUniversity Australia<br>Rockhampton QLD 4702
Organisation:	CQUniversity Australia
Area:	Centre for Physical Activity Studies
Position:	NHF & NHMRC Post-Doctoral Research Fellow
Contact	(Bus) +61(0)7 4923 2042 (AH) -<br>(Mob) - (Fax) -
Email:	c.vandelanotte@cqu.edu.au

2.2.2... Summary of qualifications and relevant expertise NS 4.8.7 NS 4.8.15
PhD qualified. Post doctoral research in the area of website-delivered interventions for physical activity and nutrition.

2.2.2... Please declare any general competing interests
Nil

2.2.2... Name the site(s) for which this principal researcher / investigator is responsible.
Greater Western Sydney and Central Queensland

2.2.3 Describe the role of the principal researcher / investigator in this project.
Oversee the project delivery, work with project officers to collect, clean and interpret data, work with the research team to determine the nature of data collected.

2.2.4 Is the principal researcher / investigator a student? No

2.2.1. Principal researcher / investigator 4

2.2.1. Name and contact details

Name:	Dr Mitch Duncan
Address:	Institute for Health and Social Science Research<br>CQUniversity Australia<br>Rockhampton QLD 4702
Organisation:	CQUniversity Australia
Area:	Centre for Physical Activity Studies
Position:	Senior Post-Doctoral Research Fellow
Contact	(Bus) +61(0)7 4930 6977 (AH) -<br>(Mob) - (Fax) -
Email:	m.duncan@cqu.edu.au

2.2.2... Summary of qualifications and relevant expertise NS 4.8.7 NS 4.8.15
PhD, BHMSc (Hons)
Has worked on several projects examining the barriers to and health benefits of engagement in physical activity. Experience in health promotion interventions and measurement of physical activity.

2.2.2... Please declare any general competing interests
Nil

2.2.2... Name the site(s) for which this principal researcher / investigator is responsible.
Greater Western Sydney and Central Queensland

2.2.3 Describe the role of the principal researcher / investigator in this project.

Work with research team. Oversee the project delivery, work with project officers to collect, clean and interpret data, work with the research team to determine the nature of data collected.

2.2.4 Is the principal researcher / investigator a student? No

2.2.1. Principal researcher / investigator 5

2.2.1. Name and contact details

Name: Dr Cristina Caperchione

Address: Faculty of Health and Social Development
University of British Columbia
Kelowna British Columbia V1V 1V7
Canada

Organisation: University of British Columbia

Area: Faculty of Health and Social Development

Position: Assistant Professor

Contact (Bus) +1 (0) 250 807-9679 (AH) -
(Mob) - (Fax) -

Email: cristina.caperchione@ubc.ca

2.2.2... Summary of qualifications and relevant expertise NS 4.8.7 NS 4.8.15
PhD qualified. Previous experience in health promotion interventions.

2.2.2... Please declare any general competing interests
Nil

2.2.2... Name the site(s) for which this principal researcher / investigator is responsible.
Greater Western Sydney and Central Queensland

2.2.3 Describe the role of the principal researcher / investigator in this project.
Work with research team to develop the web based content and determine the nature of data collected.

2.2.4 Is the principal researcher / investigator a student? No

2.2.1. Principal researcher / investigator 6

2.2.1. Name and contact details

Name: Prof William Kerry Mummery

Address: Faculty of Physical Education and Recreation
University of Alberta
Edmonton Alberta T6G 2H9
Canada

Organisation: University of Alberta

Area: Faculty of Physical Education and Recreation

Position: Dean

Contact (Bus) +1 (0) 780 492 3364 (AH) -
(Mob) - (Fax) +1 (0) 780 492 1008

Email: kerry.mummery@ualberta.ca

2.2.2... Summary of qualifications and relevant expertise NS 4.8.7 NS 4.8.15
Formal Qualifications B.Sc., M.Sc., Ph.D.
Professor of physical activity and population health with many years of research and grant experience.
Extensive experience with web-based physical activity interventions.

2.2.2... Please declare any general competing interests
Nil.

2.2.2... Name the site(s) for which this principal researcher / investigator is responsible.
Greater Western Sydney and Central Queensland

2.2.3 Describe the role of the principal researcher / investigator in this project.

Oversee the scientific direction of the study and the project delivery and work with the research team to determine the nature of the data collected.

2.2.4 Is the principal researcher / investigator a student? No

2.2.1. Principal researcher / investigator 7

2.2.1. Name and contact details

Name: Dr Richard Rosenkranz

Address: School of Biomedical and Health Sciences
University of Western Sydney
Locked Bag 1797
Penrith South DC NSW 1797

Organisation: University of Western Sydney

Area: School of Biomedical and Health Sciences

Position: Lecturer/Research Fellow in Physical Activity

Contact: (Bus) +61 (0)2 4620 3625 (AH) -
(Mob) - (Fax) +61 (0)2 4620 3710

Email: r.rosenkranz@uws.edu.au

2.2.2... Summary of qualifications and relevant expertise NS 4.8.7 NS 4.8.15

PhD in Human Nutrition, Master of Arts in Psychology, Master of Science in Kinesiology, Bachelor of Arts in Psychology/Human Development. Dr. Rosenkranz's research projects have focused on understanding the modifiable determinants of children's physical activity and dietary habits and the evaluation of multi-level interventions, based on modifiable determinants, that are designed to impact parents and children to promote physical activity and healthy eating.

2.2.2... Please declare any general competing interests

Nil.

2.2.2... Name the site(s) for which this principal researcher / investigator is responsible.

Greater Western Sydney and Central Queensland

2.2.3 Describe the role of the principal researcher / investigator in this project.

Dr. Rosenkranz will contribute to the team in several ways. He will assist with the implementation of the intervention, and work with the project officer and research assistants in Greater Western Sydney to monitor quality of data collection, storage, and analysis. Dr. Rosenkranz will assist with all phases of the research project from planning and implementation through data analysis and dissemination of findings through publication and professional presentation.

2.2.4 Is the principal researcher / investigator a student? No

2.3. Associate researcher(s) / investigator(s)

2.3.1 How many known associate researchers are there? (You will be asked to give contact details for these associate researchers / investigators at question 2.3.1.1) 0

2.3.2 Do you intend to employ other associate researchers / investigators? No

2.4. Contact

Provide the following information for the person making this application to the HREC.

2.4.1. Name and contact details

Name: Prof Gregory Kolt

Address: University of Western Sydney
Locked Bag 1797
Penrith South DC NSW 1797
Australia

Organisation: University of Western Sydney

Area: School of Biomedical and Health Sciences

Position: Head of School

Contact (Bus) +61(2) 4620 3747 (AH) -
(Mob) - (Fax) +61(2) 46203710

Email: g.kolt@uws.edu.au

2.5. Other personnel relevant to the research project

2.5.1 How many known other people will play a specified role in the conduct of this research project? 2

2.5.1... Describe the role, and expertise where relevant (e.g. counsellor), of these other personnel.

The project will be served by a project manager and website developer.
With a Bachelors degree in Health Science, the project manager will be responsible for the coordination and day to day running of the project. The website developer will be responsible for programming the web based platform used in the health promotion intervention.

2.5.2 Is it intended that other people, not yet known, will play a specified role in the conduct of this research project? Yes

2.6. Certification of researchers / investigators

2.6.1 Are there any relevant certification, accreditation or credentialing requirements relevant to the conduct of this research? No

2.7. Training of researchers / investigators

2.7.1 Do the researchers / investigators or others involved in any aspect of this research project require any additional training in order to undertake this research? No

3. RESOURCES

3.1. Project Funding / Support

3.1.1. Indicate how the project will be funded

3.1.1... Type of funding.
[Please note that all fields in any selected funding detail column (with the exception of the code) will need to be completed.]

	**External Competitive Grant**
Name of Grant / Sponsor	NHMRC
Amount of funding	896,350
Confirmed / Sought	Confirmed
Detail in kind support	In kind support from UWS and CQUniversity for staff, office space allocation, administrative and office support.
Indicate the extent to which the scope of this HREC application and grant are aligned	This application represents all research to be conducted with this grant.

3.1.1... How will you manage a funding shortfall (if any)?

The research project has received sufficient funding to sustain work to completion. All funds have been carefully distributed to ensure that key activities will be achieved in sufficient time. As this funding has been confirmed, and is adequate for the term of the research, a shortfall is not expected.

3.1.2 Will the project be supported in other ways eg. in-kind support/equipment by an external party eg. sponsor No

3.2. Duality of Interest

3.2.1 Describe any commercialisation or intellectual property implications of the funding/support arrangement.

Commercial implications are unknown and may depend on the outcome of the trial. The intervention will test the efficacy of existing Web 2.0 applications and, as such, some commercialisation could be applicable depending out trial outcomes.

3.2.2 Does the funding/support provider(s) have a financial interest in the outcome of the research? No

3.2.3 Does any member of the research team have any affiliation with the provider(s) of funding/support, or a financial interest in the outcome of the research? No

3.2.4 Does any other individual or organisation have an interest in the outcome of this research Yes

3.2.4... Indicate the interested party and describe the interest.

Organisations that may be interested in the outcomes of this research will be national, state and community health bodies (such as Queensland Health) and not for profit organisations (such as the National Heart Foundation and Diabetes Australia for example). The outcomes of this research may assist them in identifying effective measures of promoting physical activity.

3.2.5 Are there any restrictions on the publication of results from this research? No

4. PRIOR REVIEWS

4.1. Ethical review

4.1.0. Duration and location

4.1.0... In how many Australian sites, or site types, will the research be conducted? 2

4.1.0... In how many overseas sites, or site types, will the research be conducted? 0

Provide the following information for each site or site type (Australian and overseas, if applicable) at which the research is to be conducted

4.1.0...Site / Site Type 1

4.1.0... Site / Site Type Name
Online data collection

4.1.0... Site / Site Type Location
The online content will be hosted at the University of Western Sydney. Participants in the randomised controlled trial will be recruited to the study from two demographic areas; Greater Western Sydney and Central Queensland. The ecological trial will recruit participants from new registrants to the existing 10,000 steps website. Registration to this website is available to any member of the population, however, for the purposes of the study, participants must reside in Australia.

Greater Western Sydney is defined using the definition of the NSW Government's office of the Minister for Western Sydney (Auburn, Bankstown, Baukham, Blacktown, Blue Mountains, Camden, Campbelltown, Fairfield, Hawkesbury, Holroyd, Liverpool, Parramatta, Penrith, and Wollondilly local government areas (LGA)).

Central Queensland is defined as the population residing within the federal division of Capricornia.

4.1.0...Site / Site Type 2

4.1.0... Site / Site Type Name
University measurement attendance

4.1.0... Site / Site Type Location
Objective outcome measurements will be collected at UWS (Campbelltown campus) and CQUniversity Australia (Rockhampton campus).

4.1.0...Provide the start and finish dates for the whole of the study including data analysis

Anticipated start date 01/11/2010

Anticipated finish date 30/06/2014

4.1.0... Are there any time-critical aspects of the research project of which an HREC should be aware? Yes

4.1.0... Describe the time-critical aspects.
The agreement with the funding body (NHMRC) stipulates funding for 4 years commencing January 2010. The start was delayed by the transfer of this grant from CQUniversity to UWS in the first half of 2010. Preparative tasks, such as development of the web interface and data collection tools have commenced, primary data collection has not commenced.

To comply with this funding period data collection must commence by 1 July 2011.

4.1.1 To how many Australian HRECs (representing site organisations or the researcher’s / investigator's organisation) is it intended that this research proposal be submitted? 2

4.1.1...HREC 1

4.1.1... Name of HREC University of Western Sydney Human Research Ethics Committee (EC00314)

4.1.1...Provide the start and finish dates for the research for which this HREC is providing ethical review.

Anticipated start date or date range 01/11/2010

Anticipated finish date or date range 30/06/2014

4.1.1... For how many sites at which the research is to be conducted will this HREC provide ethical review? 2

4.1.1...Site 1

4.1.1... Name of site Online data collection

4.1.1... Which of the researchers / investigators involved in this project will conduct the research at this site?

Principal Researcher(s)	Associate Researcher(s)
Prof Gregory Kolt	
Prof Anthony Maeder	
Dr Corneel Vandelanotte	
Dr Mitch Duncan	
Dr Cristina Caperchione	
Prof William Kerry Mummery	
Dr Richard Rosenkranz	

4.1.1...Site 2

4.1.1... Name of site University measurement attendance

4.1.1... Which of the researchers / investigators involved in this project will conduct the research at this site?

Principal Researcher(s)	Associate Researcher(s)
Prof Gregory Kolt	
Prof Anthony Maeder	
Dr Corneel Vandelanotte	
Dr Mitch Duncan	
Dr Cristina Caperchione	
Prof William Kerry Mummery	
Dr Richard Rosenkranz	

4.1.1...HREC 2

4.1.1... Name of HREC Central Queensland University's Human Research Ethics Committee (EC00158)

4.1.1...Provide the start and finish dates for the research for which this HREC is providing ethical review.

**Anticipated start date or date range**	01/11/2010
**Anticipated finish date or date range**	30/06/2014
**4.1.1... For how many sites at which the research is to be conducted will this HREC provide ethical review?**	2

4.1.1...Site 1

4.1.1... Name of site Online data collection

4.1.1... Which of the researchers / investigators involved in this project will conduct the research at this site?

Principal Researcher(s)	Associate Researcher(s)
Prof Gregory Kolt	
Prof Anthony Maeder	
Dr Corneel Vandelanotte	
Dr Mitch Duncan	
Dr Cristina Caperchione	
Prof William Kerry Mummery	
Dr Richard Rosenkranz	

4.1.1...Site 2

4.1.1... Name of site University measurement attendance

4.1.1... Which of the researchers / investigators involved in this project will conduct the research at this site?

**Principal Researcher(s)**	**Associate Researcher(s)**
Prof Gregory Kolt	
Prof Anthony Maeder	
Dr Corneel Vandelanotte	
Dr Mitch Duncan	
Dr Cristina Caperchione	
Prof William Kerry Mummery	
Dr Richard Rosenkranz	

4.1.2 Have you previously submitted an application, whether in NEAF of otherwise, for ethical review of this research project to any other HRECs? No

4.3. Peer review

4.3.1 Has the research proposal, including design, methodology and evaluation undergone, or will it undergo, a peer review process? NS 1.2 Yes

4.3.1... Provide details of the review and the outcome. A copy of the letter / notification, where available, should be attached to this application.

The project merit and design have successfully undergone a peer review process by the NHMRC during the application phase. The letter of award from the NHMRC is attached as Appendix A.

Ethical Review Section

Summary

Applicant / Principal Researcher(s)

Prof Gregory Kolt
Head of School, School of Biomedical and Health Sciences

Prof Gregory Kolt
PhD qualified. Many years of experience in physical activity and health promotion research.

Potential conflicts of interest
Nil.

Prof Anthony Maeder
PhD qualified. Strong research and applied background in health informatics.

Potential conflicts of interest
Nil.

Dr Corneel Vandelanotte
PhD qualified. Post doctoral research in the area of website-delivered interventions for physical activity and nutrition.

Potential conflicts of interest
Nil

Dr Mitch Duncan
PhD, BHMSc (Hons)
Has worked on several projects examining the barriers to and health benefits of engagement in physical activity. Experience in health promotion interventions and measurement of physical activity.

Potential conflicts of interest
Nil

Dr Cristina Caperchione
PhD qualified. Previous experience in health promotion interventions.

Potential conflicts of interest
Nil

Prof William Kerry Mummery
Formal Qualifications B.Sc., M.Sc., Ph.D.
Professor of physical activity and population health with many years of research and grant experience. Extensive experience with web-based physical activity interventions.

Potential conflicts of interest
Nil.

Dr Richard Rosenkranz
PhD in Human Nutrition, Master of Arts in Psychology, Master of Science in Kinesiology, Bachelor of Arts in Psychology/Human Development. Dr. Rosenkranz's research projects have focused on understanding the modifiable determinants of children's physical activity and dietary habits and the evaluation of multi-level interventions, based on modifiable determinants, that are designed to impact parents and children to promote physical activity and healthy eating.

Potential conflicts of interest
Nil.

5. PROJECT

5.1. Type of Research

5.1.1 Tick as many of the following 'types of research' as apply to this project. Your answers will assist HRECs in considering your proposal. A tick in some of these boxes will generate additional questions relevant to your proposal (mainly because the National Statement requires additional ethical matters to be considered), which will appear in Section 9 of NEAF.

This project involves:

[X] Research using quantitative methods, population level data or databanks, e.g survey research, epidemiological research NS 3.2

5.1.2 Does the research involve limited disclosure to participants? NS 2.3 — No

5.1.3 Are the applicants asking the HREC / review body to waive the requirement of consent? NS 2.3.5 — No

5.2. Research plan

5.2.1 Describe the theoretical, empirical and/or conceptual basis, and background evidence, for the research proposal, eg. previous studies, anecdotal evidence, review of literature, prior observation, laboratory or animal studies (4000 character limit). NS 1.1

Physical inactivity remains one of the leading modifiable causes of death and disease in Australia. National Australian physical activity surveys indicate that less than half of the adult Australian population are sufficiently active to obtain health benefits. It has been estimated that physical inactivity contributes to more than 8,000 deaths in Australia each year, and that for every 1% of the Australian population who would become sufficiently physically active, some $7.2 million in health care costs could be saved. Novel approaches for increasing physical activity, with potential to reach broad populations at an acceptable cost, are required.

Over the past decade there has been unprecedented growth in Internet use world-wide. In 2007-2008 there were over 4.3 million Australian households with broadband Internet access (52% of all homes), a five-fold increase over the past ten years. This exponential growth has been paralleled by research into the beneficial uses of the Internet in terms of social marketing and health promotion. In the Australian context the 10,000 Steps project is an exemplar of a physical activity social marketing/health promotion program that has successfully used the Internet to extend the reach of the program beyond its initial project mandate as a whole-of-community intervention, known as 10,000 Steps Rockhampton.

We seek to test the efficacy of web 2.0 applications in the engagement and retention of, and physical activity behaviour in, people who access a publicly available physical activity promotion website (10,000 Steps). The proposal consists of two studies which seek to achieve the overall aim of the projects - a randomised control trial (RCT) and a 'real world' ecological trial (ET). The RCT will allow us to study the problem in a rigorous, controlled setting with high internal validity. The ET, by comparison, takes advantage of the unique 'real world' sample available to the research team through the existing 10,000 Steps website, which currently has over 95,000 registered users and attracts, On average, over 1,000 new registrants per month.

The approach in this application is highly novel as it tests the benefits of new generation Web 2.0 applications in terms of engagement and retention, and relates these behaviours to the outcome measure of public health interest; physical activity in a rigorous randomised control trial and in an ecologically valid setting that few researchers may ever be able to match. Outcomes of this project offer the promise of increased understanding of methods and techniques that may increase the effectiveness of the internet, a tool whose reach is expanding at a rate that is currently exceeding our grasp in terms of research into the understanding of the applications that can be effectively used to engage individuals and promote health at the population level.

5.2.2 State the aims of the research and the research question and/or hypotheses, where appropriate.

The overall aim of the project is to study the effectiveness of new generation Web 2.0 applications in physical activity health promotion.

Aim 1: To develop and test the usability of a physical activity promotion website featuring Web 2.0 applications.

Aim 2: To study the relative efficacy of web 2.0 applications in terms of physical activity behavioural change compared to an existing conventional (Web 1.0) web-based physical activity promotion site and control group.

Aim 3: To study the utility of web 2.0 applications in the engagement and retention in an ecologically valid setting through the use of web 2.0 applications within an existing, publicly available physical activity health promotion web-site.

To accomplish these aims a series of hypotheses and research questions have been established.

Randomised Control Trial

The hypotheses that will be tested are:

• There will be significantly greater engagement during the course of the study with the Web 2.0 website, as measured by website tracking software, when compared to the Web 1.0 website.
• There will be significantly greater retention on the website in the Web 2.0 arm of the study than in the conventional (Web 1.0) condition.
• Participants in the Web 2.0 condition will display higher energy expenditure (accelerometry) and higher levels of physical activity (Active Australia) at 3, 12 and 24 months post intervention than in the Web 1.0 or control condition.
.• There will be a lower decline in physical activity levels across time (within subject) in the Web 2.0 condition than in the Web 1.0 Condition or control condition.

The hypotheses relevant to the randomised control trial will be addressed by the following research questions:

1). Is there a significant change in levels of physical activity across a 3, 12 and 24 month period for participants accessing an internet-based physical activity promotion website compared to a control group?
2). Is there a significant difference in physical activity behaviour between individuals utilising a first generation (Web 1.0) site, those utilising a second generation (Web 2.0) website, and a control group?
3). Are there differences in physical activity recidivism between Web 1.0 and Web 2.0 platforms across a 12- and 24- month period?

Ecological Trial

The Hypotheses tested are:

• There will be significantly greater engagement during the course of the study with the Web 2.0 website, as measured by website tracking software, when compared to the Web 1:0 website.
• There will be significantly greater retention on the website across the course of the study in the Web 2.0 arm of the study than in the conventional (Web 1.0) condition.
• Participants in the Web 2.0 condition will display higher levels of physical activity (Active Australia Survey) at 3 months, 1 and 2-years post intervention than in the Web 1.0 condition.
• There will be a lower decline in physical activity levels across time (within subject) in the Web 2.0 condition than in the Web 1.0 Condition.

The hypotheses relevant to the ecological trial will be addressed by the following research questions:

1). Is there a significant change in levels of physical activity across a 3, 12 and 24 month period for participants accessing an internet-based physical activity promotion website?
2). Is there a significant difference in physical activity behaviour between individuals utilising a first generation (Web 1.0) site and those utilising a second generation (Web 2.0) website?
3). Are there differences in engagement and retention of participates between first generation (Web 1.0) and second generation (Web 2.0) platforms across a 12- and 24- month period?

5.2.3 Has this project been undertaken previously? No

5.3. Benefits/Risks

5.3.0 Does the research involve a practice or intervention which is an alternative to a standard practice or intervention? No

5.3.2 What expected benefits (if any) will this research have for the wider community?

This efficacy testing is an essential preliminary process for the use of web content that can be utilised successfully in engaging individuals in greater levels of physical activity. By conducting this research, content that is effective in achieving increases in physical activity can be evaluated and the longevity of resulting behaviour modification can be assessed. If the tool is found to be effective, the expected benefits of the research include an intervention that may be made available to the wider community which can increase positive health behaviours, particularly in relation to physical activity participation. This, in turn, can contribute to decreased prevalence of chronic disease associated with physical inactivity in the wider community.

5.3.3 What expected benefits (if any) will this research have for participants? NS 2.1

Participants in this research will receive health benefits through increased opportunities for physical activity. Such health benefits may include improved cardiovascular and cardio-respiratory fitness, reduced risk of chronic illness and disease, improved sleep, lower blood pressure, and decreased percentage body fat. In addition, participants have the opportunity to contribute to the development of an intervention conducted within their local community, which may be made available to the wider community in the future if found to be effective.

5.3.4 Are there any risks to participants as a result of participation in this research project? NS 2.1 — Yes

5.3.5 Explain how the likely benefit of the research justifies the risks of harm or discomfort to participants. NS 1.6

The research may expose participants to longer durations of physical activity, at levels in accordance with normal daily activity. However, participants will be encouraged to achieve 10,000 steps per day which correlates with a moderate level of physical activity and is beneficial to health. Therefore, participants should not experience greater physical stress or exertion through participation in this research. Participants may experience some inconvenience in completing online surveys and wearing Actigraph® physical activity monitors. This inconvenience is seen to be outweighed by the health benefits of participation.

5.3.8 Are there any other risks involved in this research? eg. to the research team, the organisation, others — No

5.3.9 Is it anticipated that the research will lead to commercial benefit for the investigator(s) and or the research sponsor(s)? — No

5.3.11 Is there a risk that the dissemination of results could cause harm of any kind to individual participants - whether their physical, psychological, spiritual, emotional, social or financial well-being, or to their employability or professional relationships - or to their communities? — No

5.4. Monitoring

Refer to NS 3.3.19 - 3.3.25

5.4.1 What mechanisms do the researchers / investigators intend to implement to monitor the conduct and progress of the research project? NS 5.5

The conduct and progress of this research will be monitored by the Project Steering Committee (Principal Investigators and Project Manager) which will meet monthly for the duration of the project. In addition, this research is subject to monitoring milestones by the funding body. Annual progress reports will be submitted by the project investigators and study sites have agreed to data storage and process auditing in accordance with the NHRMC guidelines.

6. PARTICIPANTS

6.1. Research participants

6.1.1 The National Statement identifies the need to pay additional attention to ethical issues associated with research involving certain specific populations.

This question aims to assist you and the HREC to identify and address ethical issues that are likely to arise in your research, if its design will include one or more of these populations. Further, the National Statement recognizes the cultural diversity of Australia's population and the importance of respect for that diversity in the recruitment and involvement of participants. Your answer to this question will guide you to additional questions (if any) relevant to the participants in your study.

6.1.1 Tick as many of the following 'types of research participants' who will be included because of the project design, or their inclusion is probable, given the diversity of Australia's population. If none apply, please indicate this below.

	**c) Design specifically excludes**
Children and/or young people (ie. <18 years)<br>NS 4.2	[X]

6.2. Participant description

6.2.1 How many participant groups are involved in this research project? 5

6.2.2 What is the expected total number of participants in this project at all sites?
3398

6.2.3. Group 1

6.2.3... Group name for participants in this group
Randomised Controlled Trial Control group

6.2.3... Expected number of participants in this group
168

6.2.3... Age range
18+

6.2.3... Other relevant characteristics of this participant group
Adults who are ambulatory and who are able to read and/or speak English but who are engaged in less than 150 minutes of moderate physical activity on 5 or more days of the week. Participants will receive a pedometer but will not have access to online content. Participants who do not successfully complete the Physical Activity Readiness Questionnaire (PAR-Q) will be referred to their GP and excluded from the study.

6.2.3... Why are these characteristics relevant to the aims of the project?
The aim of the randomised control trial is to test whether the integration of Web 2.0 applications achieves any increase in physical activity behavioural change or maintenance, when compared to the existing generation (Web 1.0) site, or to a non-intervention control group.

6.2.3. Group 2

6.2.3... Group name for participants in this group
Randomised Controlled Trial Web 1.0 group

6.2.3... Expected number of participants in this group
168

6.2.3... Age range
18+

6.2.3... Other relevant characteristics of this participant group
Adults who are ambulatory and who are able to read and/or speak English but who are engaged in less than 150 minutes of moderate physical activity on 5 or more days of the week. Participants will receive a pedometer and will have access to online content with Web 1.0 applications. Participants who do not successfully complete the Physical Activity Readiness Questionnaire (PAR-Q) will be referred to their GP and excluded from the study.

6.2.3... Why are these characteristics relevant to the aims of the project?
The aim of the randomised control trial is to test whether the integration of Web 2.0 applications achieves any increase in physical activity behavioural change or maintenance, when compared to the existing generation (Web 1.0) site, or to a non-intervention control group.

6.2.3. Group 3

6.2.3... Group name for participants in this group
Randomised Controlled Trial Web 2.0 group

6.2.3... Expected number of participants in this group
168

6.2.3... Age range
18+

6.2.3... Other relevant characteristics of this participant group
Adults who are ambulatory and who are able to read and/or speak English but who are engaged in less than 150 minutes of moderate physical activity on 5 or more days of the week. Participants will receive a pedometer and will have access to online content with Web 2.0 applications. Participants who do not successfully complete the Physical Activity Readiness Questionnaire (PAR-Q) will be referred to their GP and excluded from the study.

6.2.3... Why are these characteristics relevant to the aims of the project?
The aim of the randomised control trial is to test whether the integration of Web 2.0 applications achieves any increase in physical activity behavioural change or maintenance, when compared to the existing generation (Web 1.0) site, or to a non-intervention control group.

6.2.3. Group 4

6.2.3... Group name for participants in this group
Ecological Trial Web 1.0 group

6.2.3... Expected number of participants in this group
1447

6.2.3... Age range
18+

6.2.3... Other relevant characteristics of this participant group
Adults who are ambulatory and who are able to read and/or speak English but who are engaged in less than 150 minutes of moderate physical activity on 5 or more days of the week. Participants will have access to online content with Web 1.0 applications. Participants who do not successfully complete the Physical Activity Readiness Questionnaire (PAR-Q) will be referred to their GP and excluded from the study.

6.2.3... Why are these characteristics relevant to the aims of the project?
An ecological trial of efficacy of Web 1.0 or Web 2.0 platforms in the actual population that accesses the existing 10,000 Steps website. Users will be tracked over a period of two years to study the effects of the new generation applications on user engagement and retention.

6.2.3. Group 5

6.2.3... Group name for participants in this group
Ecological Trial Web 2.0 group

6.2.3... Expected number of participants in this group
1447

6.2.3... Age range
18+

6.2.3... Other relevant characteristics of this participant group
Adults who are ambulatory and who are able to read and/or speak English but who are engaged in less than 150 minutes of moderate physical activity on 5 or more days of the week. Participants will have access to online content with Web 2.0 applications. Participants who do not successfully complete the Physical Activity Readiness Questionnaire (PAR-Q) will be referred to their GP and excluded from the study.

6.2.3... Why are these characteristics relevant to the aims of the project?
An ecological trial of efficacy of Web 1.0 or Web 2.0 platforms in the actual population that accesses the existing 10,000 Steps website. Users will be tracked over a period of two years to study the effects of the new generation applications on user engagement and retention.

6.2.4. Your response to questions at Section 6.1 - Research Participants' indicates that the following participant groups are excluded from your research. If this is not correct please return to section 6.1 to amend your answer.

Children and/or young people (ie. <18 years)

6.2.4... Have any particular potential participants or groups of participants been excluded from this research? In answering this question you need to consider if it would be unjust to exclude these potential participants. NS 1.4

Children and young people (<18 years) have been excluded to explore the efficacy of Internet based physical activity promotion in adults. The national Australian physical activity recommendations for children are different than those for adults and our intervention materials are specifically designed for adults. Furthermore, children and young people respond differently to online technology and hence they have not been included in this research. Depending on the results of this research the online technology tested may be utilised to develop health promotion initiatives in other populations.

6.3. Participation experience

6.3.1 Provide a concise detailed description, in not more than 200 words, in terms which are easily understood by the lay reader of what the participation will involve.

All participants will be required to complete a survey of their representative physical activity patterns and regularly record their incidental physical activity levels. Participants in web-based interventions will be provided with directions and instructions for the respective web platforms and encouraged to utilise and contribute to these tools throughout the duration of the project.

Outcome measures will be collected three times over the course of the study (3 months, 12 months and 24 months), participants will be requested to present for outcome measurements (height, weight, and waist circumference) and to wear an accelerometer during waking hours for 7 days to quantify physical activity.

6.4. Relationship of researchers / investigators to participants

6.4.1 Specify the nature of any existing relationship or one likely to rise during the research, between the potential participants and any member of the research team or an organisation involved in the research.

Some participants may know, be familiar with the work of members of the research team, or be members of other community groups which the research team may be part of, however their involvement, or non-involvement with the study is voluntary and non-participation will not affect their relationship with any of these groups.

6.4.2 Describe what steps, if any, will be taken to ensure that the relationship does not impair participants' free and voluntary consent and participation in the project.

Participation or non-participation in this study will not affect a participant's relationship with the involved organisations. It will be reinforced that participation is completely voluntary and that participants have the right to withdraw at any time without penalty.

6.4.3 Describe what steps, if any, will be taken to ensure that decisions about participation in the research do not impair any existing or foreseeable future relationship between participants and researcher / investigator or organisations.

Participants will be informed that their involvement or non-involvement in this project is completely voluntary and will not influence any past, current or future relationships with the organisations involved. Participants will be informed that they are free to withdraw and cease participation in the study at any time for whatever reason. Prior to commencement of the study clarification (including written consent) will be sought to confirm that participants understand the requirements of the study and consent to participate.

6.4.4 Will the research impact upon, or change, an existing relationship between participants and researcher / investigator or organisations.? No

6.5. Recruitment

6.5.1 What processes will be used to identify potential participants?

Potential participants for the RCT will be randomly selected from postcode and invited to participate during telephone contact with a partner organisation (Population Research Laboratory at CQUniversity).

Participants for the ecological trial will be self selected from visitors to the existing 10,000 Steps website who register to participate in the 10,000 Steps program.

6.5.2 Is it proposed to 'screen' or assess the suitability of the potential participants for the study? Yes

6.5.2... How will this be done?

Participants in the RCT will be screened according to the following criteria:
• Adult (18 years and older)
• Have computer and internet access in their principle residence
• Speak and read the English language
• Ambulatory
• Fail to meet the current physical activity guidelines on 5 or more days of the week
• Usually live in a Local government area in greater western Sydney or the federal division of Capricornia
• Not be a current member, or have previously been a member, of the 10,000 Steps program

Participants in the Ecological trial will not be screened on the above criteria, however, participants in the

RCT will be ineligible to participate in the ecological trial. Eligibility will be determined during the recruitment telephone call for RCT participants. For participants in the Ecological trial, eligibility (being over 18 years of age) will be determined upon registering on the 10,000 steps website.

Participants in all study groups will have their physical condition and suitability to participate in a physical activity regimen assessed with the Physical Activity Readiness Questionnaire (PAR-Q)

6.5.3 Describe how initial contact will be made with potential participants.

Potential RCT participants will be will be randomly selected from postcode, contacted by telephone and invited to participate. This recruitment will be done by Computer-Assisted Telephone Interviewing (CATI) at the Population Research Laboratory at CQUniversity. Ecological trial participants will be self selected from visitors to the 10,000 Steps website who chose to participate. Currently the 10,000 Steps website averages 1,000 new registrations per month.

6.5.3... Do you intend to include both males and females in this study? Yes

6.5.3... What is the expected ratio of males to females that will be recruited into this study and does this ratio accurately reflect the distribution of the disease, issue or condition within the general community?

For the RCT a 1:1 gender ratio will be targeted to reflect gender distribution in the general community. The Ecological trial will not target recruits to meet a gender ratio. However, a gender ratio or 2:1 (F:M) is consistent with current registrants on the 10,000 Steps website and is expected be replicated in this study.

6.5.4 Is an advertisement, e-mail, website, letter or telephone call proposed as the form of initial contact with potential participants? Yes

6.5.4... Provide details and a copy of text/script.

Participants in the RCT will receive a telephone call inviting them to participate. A transcript of the invitation to participate in the RCT will be developed by research staff and will be provided to HREC prior to recruitment and data collection commencing. The telephone call will be through the Population Research Laboratory at CQUniversity.

Participants in the Ecological trial are selected from visitors to the 10,000 Steps website and will not receive an invitation to participate and the program will not be advertised.

6.5.5 If it became known that a person was recruited to, participated in, or was excluded from the research, would that knowledge expose the person to any disadvantage or risk? No

6.6. Consent process

6.6.1 Will consent for participation in this research be sought from all participants? Yes

6.6.1... Will there be participants who have capacity to give consent for themselves? Yes

6.6.1... What mechanisms/assessments/tools are to be used, if any, to determine each of these participant's capacity to decide whether or not to participate?

All potential participants will be screened using the inclusion/exclusion criteria and will be adults capable of providing consent to participate. Participants will be provided with a detailed information sheet to assist them in understanding the purpose, methods, risks and potential benefits of the research and in deciding to consent to participate in the study. Participants will be provided with means to correspond with the research team when deciding if they wish to be involved or not.

6.6.1... Are any of the participants children or young people? No

6.6.1... Will there be participants who do not have capacity to give consent for themselves? No

6.6.1... Describe the consent process, ie how participants or those deciding for them will be informed about, and choose whether or not to participate in, the project.

Participants will be provided with an information sheet (Appendix B) outlining the research project and what participation involves. In addition, participants will be verbally informed of the requirements of the study prior to participation. All participants will then be required to give their written consent (Appendix C) to participate in the randomised control trial.

6.6.1... If a participant or person on behalf of a participant chooses not to participate, are there specific consequences of which they should be made aware, prior to making this decision? 4.6.6 - 4.6.7

There are no consequences associated with choosing not to participate in this research. Potential participants will be invited to participate of their own volition and refusal will not prejudice future involvement with any of the organisations or individuals responsible for this research or in any other way.

6.6.1... Might individual participants be identifiable by other members of their group, and if so could this identification could expose them to risks?

This study will encourage interaction between group members to promote adherence to a physical activity

regimen. It is possible, therefore, that individual participants will be able to identify each other as a result of their involvement and/or participation in this research. However, this will not expose them to any risk.

6.6.1... If a participant or person on behalf of a participant chooses to withdraw from the research, are there specific consequences of which they should be made aware, prior to giving consent?

There are no consequences associated with withdrawing from this study. Prior to the trial commencing, participants will be informed that they can cease participating at any stage without prejudice.

6.6.1... Specify the nature and value of any proposed incentive/payment (eg. movie tickets, food vouchers) or reimbursement (eg travel expenses) to participants.

The benefits of adhering to a regular physical activity regimen will be used as an incentive to encourage participation.

Participants in the RCT will be provided with a $10 fuel voucher to cover travel costs associated with attending the objective measurement and a pedometer as part of the research. Issuing a pedometer will not be used as an incentive to participate but as an important participation tool to record daily physical activity. Participants will retain the pedometers used at the conclusion of the study.

Participants in the Ecological trial will receive no incentive to participate in this research.

6.6.1... Explain why this offer will not impair the voluntary nature of the consent, whether by participants' or persons deciding for their behalf. NS 2.2.10 - 2.2.11

It is unlikely that providing a pedometer to participants would be a successful incentive. Participation in this research may require regular commitment to physical activity over an extended period (up to two years) and will require intrinsic motivation, such as aspiring to potential health benefits. The pedometer will not be actively promoted as an incentive to participate.

There are significant time and travel demands in attending objective measurement sessions at a University campus. Subjects will be reimbursed for travel costs incurred in attending 4 objective measurement sessions over the two years of the study.

6.6.3 Do you propose to obtain consent from individual participants for your use of their stored data/samples for this research project? No

6.6.3... Give justification

This is not deemed necessary as access to stored data or samples acquired from participants prior to this research are not needed. Participant information and data will only be collected during the trial.

8. CONFIDENTIALITY/PRIVACY

8.1. Do privacy guidelines need to be applied in the ethical review of this proposal?

8.1.1 Indicate whether the source of the information about participants which will be used in this research project will involve:

[X] collection directly from the participant

8.1.1... Information which will be collected for this research project directly from the participant

8.1.1... Describe the information that will be collected directly from participants. Be specific where appropriate.

Participants will be asked to complete the following survey instruments and have the following measurements taken at each data collection time point (i.e. baseline, 3-month, 12-month and 24-month time points).

Physical activity
- Active Australia Survey Instrument
- Accelerometer - monitoring of physical activity with an accelerometer worn for 3 days

Demographic measures
- Age
- Gender
- Postcode
- Website usage statistics will also be recorded

Anthropomorphic data
- Height
- Weight
- BMI
- Waist circumference

8.1.1... The information collected by the research team about participants will be in the following form(s). Tick more than one box if applicable.

[X] individually identifiable

8.1.1... Give reasons why it is necessary to collect information in individually identifiable or re-identifiable form.

Information collected from completion of the primary and secondary outcome measures will be individually identifiable so that participant retention and compliance can be monitored across the data collection time points within the trial for efficacy comparison.

8.1.1... Consent process

You have indicated that you will be varying the conditions of or waiving consent. See questions in section 6.6

8.1.1... Will consent be specific or extended or unspecified? NS 2.2.14 - 2.2.18 Specific

8.1.1... Provide reasons why this form of consent has been chosen. You may need to revise your answer at 6.6.1.1.3 to provide details on the consent process

Data from this study will only be used for the specific purposes of this research.

8.2. Using information from participants

8.2.1 Describe how information collected about participants will be used in this project.

Outcome measures will be collated and analysed to determine the effect of Web 2.0 applications on physical activity in comparison to an existing Web 1.0 platform and the control group. This analysis will allow researchers to identify whether the intervention has been effective within the target group (i.e. if the intervention has increased physical activity levels in the target group). Outcomes of this research will be published in peer reviewed literature and other scientific reports, and presented at appropriate academic and other conferences.

8.2.2 Will any of the information used by the research team be in identified or re-identifiable (coded) form? Yes

8.2.2... Indicate whichever of the following applies to this project:

[X] Information collected for, used in, or generated by, this project will not be used for any other purpose.

8.2.4 List ALL research personnel and others who, for the purposes of this research, will have authority to

use or have access to the information and describe the nature of the use or access. Examples of others are: student supervisors, research monitors, pharmaceutical company monitors .

Only the research team and other associated personnel listed within this application will have access to identifiable data. This is necessary for the analysis and interpretation of the data and to develop reports or publications arising from the research.

8.3. Storage of information about participants during and after completion of the project

8.3.1 In what formats will the information be stored during and after the research project? (eg. paper copy, computer file on floppy disk or CD, audio tape, videotape, film)

Data will be collected in both electronic (computer files) and written format. Data will be stored in both hard (paper) copy and soft (electronic) copy format.

8.3.2 Specify the measures to be taken to ensure the security of information from misuse, loss, or unauthorised access while stored during and after the research project? (eg. will identifiers be removed and at what stage? Will the information be physically stored in a locked cabinet?)

Paper documents will be stored in a locked cabinet and secured offices of the researchers and other personnel relevant to the project. Electronic files will be stored on password-protected computers. Hard copy data will not be allowed off site.

Given the this research involves a proposed waiver of consent and the intent of exposing illegal activity [see NS 4.6.1] the HREC must be satisfied that your response to this question has justified that there is sufficient protection of the privacy of the participants.

8.3.5 The information which will be stored at the completion of this project is of the following type(s). Tick more than one box if applicable.

[X] non-identifiable

8.3.6 For how long will the information be stored after the completion of the project and why has this period been chosen?

Data will be stored for a minimum of five years as recommended by the Records Management Policy of the University of Western Sydney.

8.3.7 What arrangements are in place with regard to the storage of the information collected for, used in, or generated by this project in the event that the principal researcher / investigator ceases to be engaged at the current organisation?

Data will be retained by UWS. In the situation where the principal researcher/s cease to be engaged by UWS, the information will be transferred to another Researcher at UWS in accordance with the UWS Research Code of Practice. This data will remain securely stored under the conditions previously specified in 8.3.2.

8.4. Ownership of the information collected during the research project and resulting from the research project

8.4.2 Who is understood to own the information resulting from the research, eg. the final report or published form of the results?

The intellectual property developed as a result of this research will be shared in proportion to the agreed inventive contribution of each party as per the executed Collaborating Institutions Agreement.

8.4.3 Does the owner of the information or any other party have any right to impose limitations or conditions on the publication of the results of this project? No

8.5. Disposal of the information

8.5.1 Will the information collected for, used in, or generated by this project be disposed of at some stage? Yes

8.5.1... At what stage will the information be disposed?

The information may be disposed of a minimum of five (5) years after the last publication from the data in this research. A copy of the original data will be retained by the chief investigator in accordance with University of Western Sydney Research Code of Practice

8.5.1... How will information, in all forms, be disposed?

Both hard and electronic copies of the data will be disposed of in accordance with the records management policy of the University of Western Sydney.

8.6. Reporting individual results to participants and others

8.6.1 Is it intended that results of the research that relate to a specific participant be reported to that participant? No

8.6.1... Explain/justify why results will not be reported to participants.

Participants will not be provided with their individual results. However, a plain English summary report of results of the study, including themes found in all trial groups, will be made available to all participants at the

conclusion of the study.

8.6.2 Is the research likely to produce information of personal significance to individual participants? Yes

8.6.3 Will individual participant's results be recorded with their personal records? No

8.6.4 Is it intended that results that relate to a specific participant be reported to anyone other than that participant? No

8.6.5 Is the research likely to reveal a significant risk to the health or well being of persons other than the participant, eg family members, colleagues No

8.6.6 Is there a risk that the dissemination of results could cause harm of any kind to individual participants - whether their physical, psychological, spiritual, emotional, social or financial well-being, or to their employability or professional relationships - or to their communities? No

8.6.7 How is it intended to disseminate the results of the research? eg report, publication, thesis

The results of the research will be published in a plain English summary of the findings and in a final report to the funding body. The results of the research will be presented to academic and non-academic audiences by way of conference presentations as well as academic and non-academic publications, including reports and journal articles.

8.6.8 Will the confidentiality of participants and their data be protected in the dissemination of research results? Yes

8.6.8... Explain how confidentiality of participants and their data will be protected in the dissemination of research results

Results from the outcome measure will not be presented in a way that adversely affects the confidentiality of participants. Findings from the process outcomes will only include generalised themes acquired from the responses of all participants. The description of participants will not allow identification of individual participants, and individual results and individual names will not be revealed. Final reports and publications will only consist of aggregated results.

9. DECLARATIONS AND SIGNATURES

9.1 Project Title

WALK 2.0: Investigating the internal and external validity of Web 2.0 applications in promoting physical activity

9.2 Human Research Ethics Committee to which this application is made

Central Queensland University's Human Research Ethics Committee (EC00158)

University of Western Sydney Human Research Ethics Committee (EC00314)

9.3 Signatures and undertakings

Applicant / Principal Researchers (including students where permitted)

I/we certify that:
- All information is truthful and as complete as possible.
- I/we have had access to and read the National Statement on Ethical Conduct in Research Involving Humans.
- the research will be conducted in accordance with the National Statement.
- the research will be conducted in accordance with the ethical and research arrangements of the organisations involved.
- I/we have consulted any relevant legislation and regulations, and the research will be conducted in accordance with these.
- I/we will immediately report to the HREC anything which might warrant review of the ethical approval of the proposal NS 5.5.3 including:
    - serious or unexpected adverse effects on participants;
    - proposed changes in the protocol; and
    - unforseen events that might affect continued ethical acceptability of the project.
- I/we will inform the HREC, giving reasons, if the research project is discontinued before the expected date of completion NS 5.5.6 see NS 5.5.8(b);
- I/we will adhere to the conditions of approval stipulated by the HREC and will cooperate with HREC monitoring requirements. At a minimum annual progress reports and a final report will be provided to the HREC.

Applicant / Chief Researcher(s) / Principal Researcher(s)

Prof Gregory Kolt<br>University of Western Sydney	[signature]<br>Signature	2/11/10<br>Date
Prof Gregory Kolt<br>University of Western Sydney	[signature]<br>Signature	2/11/10<br>Date
Prof Anthony Maeder<br>University of Western Sydney	[signature]<br>Signature	2/11/2010<br>Date
Dr Corneel Vandelanotte<br>CQUniversity Australia	[signature]<br>Signature	2/11/2010<br>Date
Dr Mitch Duncan<br>CQUniversity Australia	[signature]<br>Signature	2/11/2010<br>Date
Dr Cristina Caperchione<br>University of British Columbia	Signature	/ /<br>Date
Prof William Kerry Mummery<br>University of Alberta	Signature	/ /<br>Date
Dr Richard Rosenkranz<br>University of Western Sydney	[signature]<br>Signature	2/11/2010<br>Date

Heads of departments/schools/research organisation

I/we certify that:
- I/we are familiar with this project and endorse its undertaking;
- the resources required to undertake this project are available;
- the researchers have the skill and expertise to undertake this project appropriately or will undergo appropriate training as specified in this application.

__________ ____________________ ____________________________________
Title First name Surname

____________________________________ ____________________________________
Position Organisation name

____/____/____ ____________________________________
Date Signature

10. ATTACHMENTS

This page and all pages that follow don't need to be submitted to your HREC.

10.1 List of Attachments

Core Attachments	Attachments which may be required/appropriate.
Recruitment/invitation	Copy of advertisement, letter of invitation etc
Participant Information	Copy or script for participant<br>Copy or script for parent, legal guardian or person responsible as appropriate
Consent Form	Copy for participant<br>For parent, legal guardian or person responsible as appropriate<br>For, optional components of the project eg. genetic sub study
Peer review	Copy of peer review report or grant submission outcome
HREC approvals	Copy of outcome of other HREC reviews

Attachments specific to project or participant group	Attachments which may be required/appropriate.
Epidemiological research	Evidence of support/permission from database custodian for proposed access / use of data
Children and/or young people (ie. <18 years)	Information/consent form for parent, legal guardian or person responsible

10.2 Participant information elements
Core Elements
Provision of information to participants about the following topics should be considered for all research projects.

Core Elements	Issues to consider in participant information
About the project	Full title and / or short title of the project<br>Plain language description of the project<br>Purpose / aim of the project and research methods as appropriate<br>Demands, risks, inconveniences, discomforts of participation in the project<br>Outcomes and benefits of the project<br>Project start, finish, duration
About the investigators / organisation	Researchers conducting the project (including whether student researchers are involved)<br>Organisations which are involved / responsible<br>Organistions which have given approvals<br>Relationship between researchers and particpants and organisations
Participant description	How and why participants are chosen<br>How participants are recruited<br>How many participants are to be recruited
Participant experience	What will happen to the particant, what will they have to do, what will they experience?<br>Benefits to individual, community, and contribution to knowledge<br>Risks to individual, community<br>Consequences of participation
Participant options	Alternatives to participation<br>Whether participation may be for part of project or only for whole of project<br>Whether any of the following will be provided: counselling, post research follow-up, or post research access to services, equipment or goods
Participants rights and responsibilities	That participation is voluntary<br>That participants can withdraw, how to withdraw and what consequences may follow<br>Expectations on participants, consequences of non-compliance with the protocol<br>How to seek more information<br>How to raise a concern or make a complaint
Handling of information	How information will be accessed, collected, used, stored, and to whom data will be disclosed<br>Can participants withdraw their information, how, when<br>Confidentiality of information<br>Ownership of information<br>Subsequent use of information<br>Storage and disposal of information
Unlawful conduct	Whether researcher has any obligations to report unlawful conduct of participant
Financial issues	How the project is funded<br>Declaration of any duality of interests<br>Conspensation entitlements<br>Costs to participants<br>Payments, reimbursements to participants<br>Commercial application of results
Results	What will particpants be told, when and by whom<br>Will individual results be provided<br>What are the consequences of being told or not being told the results of

Core Elements	Issues to consider in participant information
	research<br>How will results be reported / published<br>Ownership of intellectual property and commercial benefits
Cessation	Circumstances under which the participation of an individual might cease<br>Circumstances under which the project might be terminated

Research Specific Elements
Provision of information to participants about the following topics should be considered as may be relevant to the research project.

Appendix A

NHMRC award letter

Australian Government
National Health and Medical Research Council
NHMRC

In reply please quote: NHMRC Project Grant Application 589903

Prof William Kerry Mummery
Associate Dean, College of Health & Human Services
Central Queensland University
Building 18, Bruce Highway
Rockhampton QLD 4702

Dear Prof Mummery

Project Grant Application: 589903
Scientific Title: WALK 2.0: Investigating the internal and external validity of Web 2.0 applications in promoting physical activity

I am pleased to advise that the Minister for Health and Ageing, the Hon Nicola Roxon MP, has approved funding for your National Health & Medical Research Council (NHMRC) Project Grant to commence in 2010.

Approved Budget
The Approved Budget for this Project Grant is $896,350 over a period of 4 years. This budget (and its individual components) was determined by the Grant Review Panel (GRP) during its detailed assessment of the application. You should note that this budget (excluding any equipment component) is provided as a one-line grant and grantees may expend the funds as necessary to support the research project provided that:

1. all expenditure is an accordance with the requirements of the *Deed of Agreement – Research Funding Schemes* (the Deed), noting that use of funding for some purposes is expressly excluded in the Deed;
2. funding approved for specific pieces of *Equipment* is used for this purpose;
3. funding is not used to provide infrastructure that should be provided by the institution; and
4. annual financial reports itemise expenditure against outgoings, including *Salaries, Equipment* and *Direct Research Costs.*

The following table indicates the total amount in each year for which funding has been approved. These amounts are exclusive of any GST or annual indexation that may apply.

Year	**2010**	**2011**	**2012**	**2013**	**2014**
Personnel Support Packages	$219,250	$220,550	$220,550	$141,000	$
Direct Research Costs	$50,000	$20,000	$20,000	$5,000	$
Equipment	$	$	$	$	$
TOTAL	$269,250	$240,550	$240,550	$146,000	$

Outcome of the Assessment
Please find enclosed a final assessment report, which is designed to assist applicants in understanding where their application could have been made more competitive in relation to applications that were ranked more highly.

Grant Advisory Group 5 (GAG) rated your application in Category 6, when assessed against the totality of the category descriptors. Further information on the category descriptors is provided in Attachment B to the *NHMRC Project Grants Funding Policy for funding commencing in 2010* available at http://www.nhmrc.gov.au/grants/apply/projects/index.htm#pol. The membership and fields of research covered by each GAG can also be found at http://www.nhmrc.gov.au/grants/rounds/projects/index.htm.

Accepting this offer
The formal offer of grant funding will be made as part of the *Deed of Agreement – Research Funding Schemes* (the Deed) between the Australian Government and your Administering Institution. It is the responsibility of your Administering Institution to inform you of the requirements under the Deed, which sets out the amount and duration of funding, conditions or milestones, co-funding and reporting requirements. A copy of the generic Deed of Agreement is posted on the NHMRC web site for your information at: http://www.nhmrc.gov.au/grants/admin/deeds.htm

If you wish to accept the offer of funding, please contact your Research Administration Officer (RAO). Your Institution has until 31 January 2010 to advise NHMRC of your acceptance or the offer may be withdrawn. Prior to commencement of payments for this grant, your RAO is required to provide notification that all necessary clearances have been obtained.

Please direct all queries regarding this offer via your RAO as they are NHMRC's first point of contact when disseminating instructions and information about the scheme.

Participation in NHMRC Peer Review
Grant recipients are reminded that subsection 13.4 of the Deed states:

> *"The Commonwealth may request the Institution to make available to the Commonwealth, the services of Chief Investigators for the purposes of reviewing or assessing applications made under the NHMRC Funding Schemes during the Period of Funding, and the Institution will use its best endeavours to facilitate compliance by the Chief Investigator(s)."*

Accordingly, all Chief Investigators are requested to complete the *NHMRC Peer Review Participation Form* located at: http://www.nhmrc.gov.au/grants/peer/participation.htm. Your RAO will be requested to confirm completion of the *Peer Review Participation Form* by all Chief Investigators prior to returning the completed Schedule to NHMRC.

Lastly, I would like to congratulate you on your successful application and to take this opportunity to wish you well with this research project.

Yours sincerely

[Authorised for Electronic Transmission]

Michael Nutt
A/g Executive Director
Research Investment Branch
8 October 2009

cc: Research Administration Officer, Central Queensland University (CQU)

ENC: Grant Review Panel (GRP) Final Report

THE HON NICOLA ROXON MP

Minister for Health and Ageing

MEDIA RELEASE

date NR09/

$896,000 HEALTH AND MEDICAL RESEARCH FUNDING FOR CENTRAL QUEENSLAND UNIVERSITY

The Australian Government is providing $896,350 in new health and medical research funding to the Central Queensland University.

The funding, part of a $500 million government investment into the future health and wellbeing of all Australians, will be provided through the National Health and Medical Research Council.

NHMRC is the Australian Government's peak funding body for health and medical research. It selects projects for funding from among a large number of applications.

This funding will enable researchers at the university to contribute to the Government's health reform agenda to improve the health of all Australians.

The research information produced will enable us to better understand, tackle and even prevent chronic disease and other health problems in the future.

The largest slice of the overall funding has gone to Project Grants, which support individuals and teams conducting research into all areas of health. Central Queensland University has received one project grant:

WALK 2.0; using the web to promote physical activity More than half of Australians do not get enough exercise to achieve health benefits, such as reducing obesity and chronic illness. This research will look at internet based programs that have the potential to reach large numbers of people to promote physical activity, and assesses program effectiveness. The research will be carried out by Prof William Mummery and his team, with funding of $896,350.

This round of funding also includes NHMRC's Project Grant, Enabling Grant, Partnership for Better Health Grant, Standard Equipment Grant, Fellowship and Career Development Award schemes.

For more information on the funding schemes, and a full list of successful projects, visit www.nhmrc.gov.au

Media contacts: Minister Roxon's office, 02 6277 7220
Carolyn Norrie, NHMRC, 0422 008 512 or 02 6217 9190

Appendix B

Participant information sheet

(DRAFT) PARTICIPANT INFORMATION SHEET

Using web 2.0 applications to promote health-related physical activity

Thank you for your interest in participating in this research project, conducted by researchers from the University of Western Sydney (UWS), and CQUniversity (CQU) Australia. If you have any questions while reading this information sheet please do not hesitate to contact the project manager or one of the Investigators.

Who is conducting the study?

Professor Gregory Kolt, *Head of school, School of Biomedical & Health Sciences*, UWS

Professor Anthony Maeder, *Professor of Health Informatics, School of Computing & Informatics*, UWS

Dr Corneel Vandelanotte, *NHF & NHMRC Post-Doctoral Research Fellow, Institute for Health & Social Science Research*, CQU

Dr Mitch Duncan, *Senior Post-Doctoral Research Fellow, Institute for Health & Social Science Research*, CQU

Dr Cristina Caperchione, *Assistant Professor, Faculty of Health & Social Development*, University of British Columbia

Professor Kerry Mummery, *Professor & Dean, Faculty of Physical Education & Recreation*, University of Alberta

Dr Ric Rosenkranz, *Lecturer & Research Fellow School of Biomedical & Health Sciences*, UWS

You are invited to participate in a research project assessing the effectiveness and usability of a web based health promotion tool. Physical inactivity continues to contribute to ill health by increasing the risk of obesity, heart disease and diabetes. Incidental activity, such as walking, can have significant healthy benefits and programs such as 10000 steps. Access to the internet is widespread and presents an ideal opportunity to deliver this information as people can access it at any time at their own convenience. It also allows people to monitor their behaviours with ease.

Our research team has developed the Walk2.0 Project to try to improve the physical activity habits of Australian Adults. Walk2.0 will allow you to monitor your physical activity habits to encourage you to improve your physical activity routine. We would like you to participate in Walk2.0 to determine its effectiveness to improve health.

Walk2.0 will provide you with access to the Walk2.0 websites or material to monitor and record your physical activity. Because you record the activity, you can compare with your friends to see who can make the biggest and longest lasting changes to their health. The website applications and printed recording material contain the same information, it s simply different ways for you to access the information and track the changes in your behaviours.

To participate you need to be aged over 18, living in either the Central Queensland or Western Sydney regions, and have access to the internet.

If you agree to participate you will be randomly assigned to one of three groups:

A. The Walk2.0 existing website group
B. The Walk2.0 interactive website group
C. The Walk2.0 control group.

We need people in each of the three groups so that we can determine how effective each approach is to improve your health.

What would I have to do?

The University of Western Sydney
Participant information and consent forms
WALK 2.0 PROJECT

University of Western Sydney
Bringing knowledge to life

The Walk2.0 project is a 24 month project and to participate you need to be willing to increase your physical activity and to record this information. We ask you to measure your physical activity by wearing a pedometer, a small device that records the number of steps you take, and recording the steps that you take each day. All participants in the project will be supplied with a pedometer as part of the research which will remain yours to keep.

If you decide to participate we will ask you to complete a short (30 minute) survey to help us determine how effective the Walk2.0 intervention is. We will ask you to do this at the beginning of the project, three months after you ve started, twelve months after you started and, finally, 2 years after you started. These surveys will ask you some background information about yourself and also your physical activity level.

You will also be asked to attend a university measurement session at the start of the project and at 3, 12 and 24 months. During the measurement session we will measure your height, weight and waist circumference. Each measurement session will take 15 minutes and we will provide you with a $10 fuel voucher to assist with travel costs to attend the session. At the end of the measurement session we will provide you with an activity meter to wear for the following five days. This is a small battery operated device attached to a belt that measures periods of movement to tell us how much physical activity you have been doing. We will provide you with detailed information on this activity monitoring during the measurement session and a reply paid envelope to return the activity monitor to the university. You will receive your fuel voucher once the activity meter has been returned.

Will the study benefit me?

We cannot and do not promise that you will receive any benefits from participating in this study. However, a desired outcome of this research is increased physical activity levels among participants. Increased levels of physical activity may reduce your risk of developing diseases such as diabetes, cardiovascular disease and overweight and obesity

Will the study involve any risks or discomfort for me?

Because you are asked to regularly record your physical activity there may be a minimal time cost (between 2 to 5 minutes) to enter your data. However, there is no minimum requirement for time to be spent interacting with the website, the amount of time that you spend is up to you. There are no health related risks associated with participating in this research. While we are asking you to walk more often, you should continue to walk at a pace that you re comfortable with. You may encounter environmental hazards when walking and you might experience some muscle soreness as you commence in the study, especially if you have generally not been active previously. However, these risks are seen to occur uncommonly or be generally outweighed by the benefits of physical activity.

Will anyone else know the results? How will the results be disseminated?

If you consent to participate in this project by signing this document, any information that is collected through this project and that can be identified with you will be confidential. The information you provide and any measurements taken will be stored in locked filing cabinets and in password protected files so only research staff will have access to the information. we plan to publish the results in scientific literature and provide reports to interested parties. Information from this research will also be available in the form of a plain English statement of the summarised findings to project participants. In any publication or report, information will be provided in such a way that you cannot be identified

Can I withdraw from the study?

Participation is entirely voluntary: you are not obliged to participate and if you choose to participate you can withdraw at any time by returning the revocation of consent form to the Walk2.0 project office. You do not have to give a reason for withdrawing from the study and changing your mind will not affect your relationship with the researchers, other relevant research personnel, or with the universities and partner organisations conducting the research.

The University of Western Sydney
Participant information and consent forms
WALK 2.0 PROJECT

University of Western Sydney
Bringing knowledge to life

What we would like you to do:

If you agree to participate in the research we would like you to:

- Sign the consent form giving your consent to participate in the research.
- Participate in data collection at baseline, 3 month, 12 month and 24 month time points for the trial, and wear a physical activity monitoring device if asked for five days for each of the same time intervals.
- Increase your physical activity levels.
- Record your physical activity and dietary habits on either the Walk2.0 website or print materi-als.

What if I require further information?

If you have any questions regarding the project, please do not hesitate to contact the Project Officer, Trevor Savage on (02) 4620 3698 or email Walk20@uws.edu.au or any of the project s investigators.

What if I have a complaint?

This study has been approved by the University of Western Sydney Human Research Ethics Commit-tee. The Approval number is [approval number]. If you have any complaints or reservations about the ethical conduct of this research, you may contact the Ethics Committee through the Office of Re-search Services on
Tel (02) 4736 0883
Fax (02) 4736 0013 or
Email humanethics@uws.edu.au.

Thanks again for your interest in this project.

University of Western Sydney
Bringing knowledge to life

Revocation of Consent

"Using Web 2.0 applications to promote health-related physical activity"

(Only to be completed when **withdrawing** from the project)

I hereby wish to withdraw my consent to participate in the research proposal described above and I understand that such a withdrawal WILL NOT affect my relationship with the researchers, other relevant research personnel, or with the universities and partner organisations conducting this research.

____
Signature of participant

____
Name of participant

____/____/___
Date

____
Signature of witness

____
Name of witness

____/____/___
Date

Appendix C

Participant consent form

Participant consent form

I ____ consent to take part in the research project titled

“Using Web 2.0 applications to promote health-related physical activity”

I acknowledge that:

I have read the participant information sheet and I have been given the opportunity to discuss the information and my involvement in the project with the researcher/s.

The procedures required for the project and the time involved have been explained to me, and any questions I have about the project have been answered to my satisfaction.

I consent to participate in this study. I understand that I am required to attend an objective measurement session on four occasions over the course of the study and that I will receive a fuel voucher as compensation for my travel costs. I also consent to participate in activity monitoring over the course of the study and to the administration of surveys to collect information about my health and wellbeing.

I understand that my involvement is confidential and that the information gained during the study may be published but no information about me will be used in any way that reveals my identity.

I understand that I can withdraw from the study at any time, without affecting my relationship with the researcher/s now or in the future.

Signed: __________________________________

Full name: ________________________________

Date: ____/_____/____

I would like to receive a plain English statement of the findings: Yes No
I would like to be contacted in the future about other research projects: Yes No
CONTACT DETAILS (post, phone and email):

Appendix D

Statement of Compliance

with NSW Privacy Legislation
